# Amyloid aggregates induced by the p53-R280T mutation lead to loss of p53 function in nasopharyngeal carcinoma

**DOI:** 10.1038/s41419-024-06429-8

**Published:** 2024-01-11

**Authors:** Jingzhi Li, Ming Guo, Lin Chen, Zhuchu Chen, Ying Fu, Yongheng Chen

**Affiliations:** 1 Department of Oncology, NHC Key Laboratory of Cancer Proteomics & State Local Joint Engineering Laboratory for Anticancer Drugs, National Clinical Research Center for Geriatric Disorders, Xiangya Hospital, Central South University, Changsha, 410008 Hunan China; 2 Department of Obstetrics, Xiangya Hospital, Central South University, Changsha, 410008 Hunan China; 3 Molecular and Computational Biology Program, Department of Biological Sciences and Department of Chemistry, University of Southern California, Los Angeles, CAL 90089 USA

**Keywords:** Head and neck cancer, Medical research, Oncogenes

## Abstract

Nasopharyngeal carcinoma (NPC) is a malignant tumor that is highly prevalent in Southeast Asia, especially in South China. The pathogenesis of NPC is complex, and genetic alterations of tumor suppressors and proto-oncogenes play important roles in NPC carcinogenesis. p53 is unexpectedly highly expressed in NPC and possesses an uncommon mutation of R280T, which is different from a high frequency of hotspot mutations or low expression in other tumors. However, the mechanism of p53 loss of function and its correlation with R280T in NPC are still unclear. In this study, p53 amyloid aggregates were found to be widespread in NPC and can be mainly induced by the R280T mutation. Aggregated p53-R280T impeded its entry into the nucleus and was unable to initiate the transcription of downstream target genes, resulting in decreased NPC cell cycle arrest and apoptosis. In addition, NPC cells with p53-R280T amyloid aggregates also contributed aggressively to tumor growth in vivo. Transcriptome analysis suggested that p53 amyloid aggregation dysregulated major signaling pathways associated with the cell cycle, proliferation, apoptosis, and unfolded protein response (UPR). Further studies revealed that Hsp90, as a key molecular chaperone in p53 folding, was upregulated in NPC cells with p53-R280T aggregation, and the upregulated Hsp90 facilitated p53 aggregation in turn, forming positive feedback. Therefore, Hsp90 inhibitors could dissociate p53-R280T aggregation and restore the suppressor function of p53 in vitro and in vivo. In conclusion, our study demonstrated that p53-R280T may misfold to form aggregates with the help of Hsp90, resulting in the inability of sequestered p53 to initiate the transcription of downstream target genes. These results revealed a new mechanism for the loss of p53 function in NPC and provided novel mechanistic insight into NPC pathogenesis.

## Introduction

Nasopharyngeal carcinoma (NPC) is an epithelial carcinoma that originates within the mucosa of the nasopharynx [1]. In 2020, 133 000 new cases of NPC were diagnosed worldwide, accounting for 0.7% of all cancer cases. NPC exhibits a distinct geographic distribution, with a significantly higher prevalence in southern China and southeast Asia [2]. In these regions, the incidence of NPC is approximately 25 cases per 100 000 people, which is approximately 25 times higher than that in other areas [3]. The initiation and progression of NPC is a complex process involving multiple genes [4].

p53, known as the guardian of the genome, plays a crucial role in suppressing the occurrence and progression of various tumors [5]. More than half of human tumors carry p53 mutations, with a frequency exceeding 42% [6]. However, in NPC, the frequency of p53 mutations is only 30% [7], among which hotspot mutations in the DBD of p53, such as R248, R175, and R273, are not commonly observed [8]. Instead, a less recognized mutation at codon site 280 from AGA to ACA (R280T) is implicated in approximately 10% of NPC cases [9]. The functional effects and underlying mechanisms associated with the R280T mutation have received limited research attention. Although p53 expression in NPC tends to be relatively high [10, 11], it is a notable concern that p53 fails to exert substantial suppression in NPC despite its abundant presence.

Prion diseases are common in the nervous system and are thought to be caused by protein misfolding and aggregation [12]. Recent studies have suggested that tumors are also a prion disease [13, 14] induced by pathological aggregation of specific tumor suppressor proteins, notably p53 [15–19]. Several studies have explored p53 amyloid aggregation in tumors and found that multiple domains of p53 are involved in this process. The aggregation-nucleating segment (ANS), among the hydrophobic center of the DBD domain of p53 (residues 251 to 257), was confirmed as a critical region of p53 aggregation, both in vitro and in vivo [20]. Other studies have shown that destabilized p53 hotspot mutants may cause p53 aggregation independently [21–24]. The formation of mutant p53 aggregates ultimately leads to loss-of-function (LoF), gain-of-function (GoF), or dominant-negative effects (DNs) and is involved in carcinogenesis [25–27].

Although p53 aggregation has been implicated in numerous tumors, there are no reports about its involvement in NPC or the role of the R280T mutation in p53 aggregation. The R280 site is crucial for p53 due to its essential role in p53 folding and interacting with chaperone proteins. On the one hand, the cationic arginine at the 280 site, the so-called “gate-keeper”, employs its charge to repel protein aggregation [28], and when it is replaced by threonine, the risk of p53 aggregation may increase. On the other hand, crystal structure analysis and nuclear magnetic resonance demonstrated that R280 directly participates in the interaction between p53 residues and the middle domain of Hsp90 [29, 30], which is vital for proper p53 folding [31]. Thus, we speculated that the R280 mutant of p53 or abnormal interaction with Hsp90 might assist in the misfolding of p53 in NPC and accelerate p53 accumulation in the cytoplasm, ultimately resulting in the loss of p53 function in NPC.

In this study, we first found that aggregated p53 amyloid formation was widespread in NPC. Then, we revealed that the R280T mutation might be the primary driver of p53 aggregation in NPC. Specifically, the R280T mutation of p53 misaligned its proper binding with Hsp90, promoting the formation of p53 amyloid. We further validated our hypothesis by introducing Hsp90 inhibitors, which could disrupt the interaction between p53 and Hsp90 and partly restore the suppressive function of p53. In addition, transcriptomics analysis revealed changes in related essential signaling pathways or target genes expression induced by p53 aggregation. All these findings contribute to our understanding of p53 in NPC oncogenesis and progression.

## Results

### Aggregated p53 amyloid formation is widespread in NPC

**Fig. 1:**
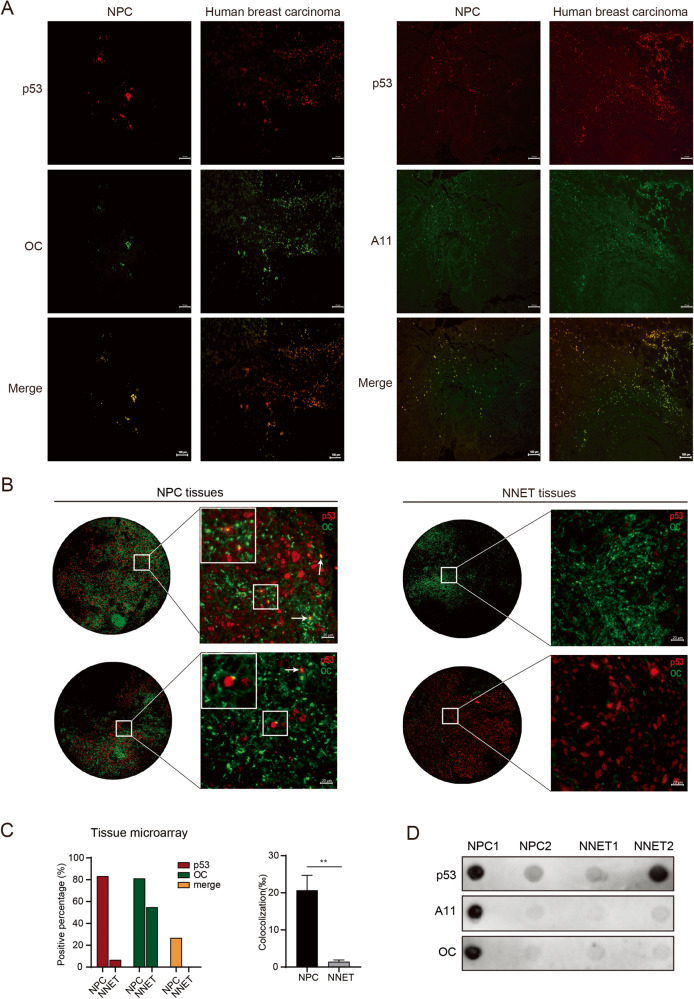
Aggregated p53 amyloid is widespread in NPC. **A** Immunofluorescence showed the amyloid and oligomer states of p53 aggregation in NPC tissues. Red represents p53 staining, and green represents OC (specific to amyloids) or A11 (specific to oligomers) antibody staining. Human breast carcinoma tissues were used as the positive control for p53 in the amyloid or oligomer state. Scale bars, 100 μm. **B** Representative staining results of p53 and OC antibodies for NPC and NNET tissue in the tissue microarray. The white arrows show the merged signals of p53 and OC antibody staining, representing amyloid aggregation of p53 in NPC tissues. Scale bars, 20 μm. **C** Statistical analysis of the p53 amyloid state and colocalization ratio for NPC and NNET tissues in the tissue microarray. Data are represented as mean ± SD. The *P* value was measured by the two-tailed Student’s unpaired *t* test. **P < 0.01. **D** Dot blot results for TAFs extracted from NPC and NNET tissues. The positive immunoreactivity of OC and p53 confirmed the amyloid nature of fibrils for p53 in NPC. NPC nasopharyngeal carcinoma, NNET normal nasopharyngeal epithelial tissues, TAF tissue amyloid fraction.

To examine aggregated p53 in NPC, an OC (amyloid-specific) antibody and an A11 (oligomer-specific) antibody were used for immunofluorescence staining of NPC tissues. The results showed that there were strong signals of OC staining or several signals of A11 (green), colocalizing with p53 (red) in NPC tissues (Fig. 1A). Human triple-negative breast carcinoma tissues were used as the positive control [15, 32]. To further identify the frequency of p53 aggregation in NPC, immunofluorescence assays were performed for the tissue microarray containing 31 normal nasopharyngeal epithelial tissues (NNET) and 90 NPC samples (Supplementary Fig. 1A). The results showed that 2 of 31 NNET samples (6.5%) and 75 of 90 NPC samples (83.3%) were p53 positive. Quantitative analysis revealed that p53 colocalized with the OC antibody in 20 of 75 p53-positive NPC samples (26%), and no colocalization was observed in NNET samples (Fig. 1B, C and Supplementary Fig. 1B). These results indicated that p53 amyloid aggregation was prevalent in p53-positive NPC tissues.

Furthermore, the tissue amyloid fraction (TAF) from NPC and NNET samples was extracted, and dot blot assays were performed to confirm the presence of p53 within TAF fibrils. The results showed positive immunoreactivity of the OC and A11 antibodies when the p53 antibody was positive in most NPC samples but almost negative immunoreactivity when the p53 antibody was positive in NNET samples (Fig. 1D and Supplementary Fig. 1C). All these results suggested that p53 forms amyloid fibrils in NPC tissues but not in NNET.

### p53-R280T is extensive in NPC cells and promotes p53 amyloid aggregates

**Fig. 2:**
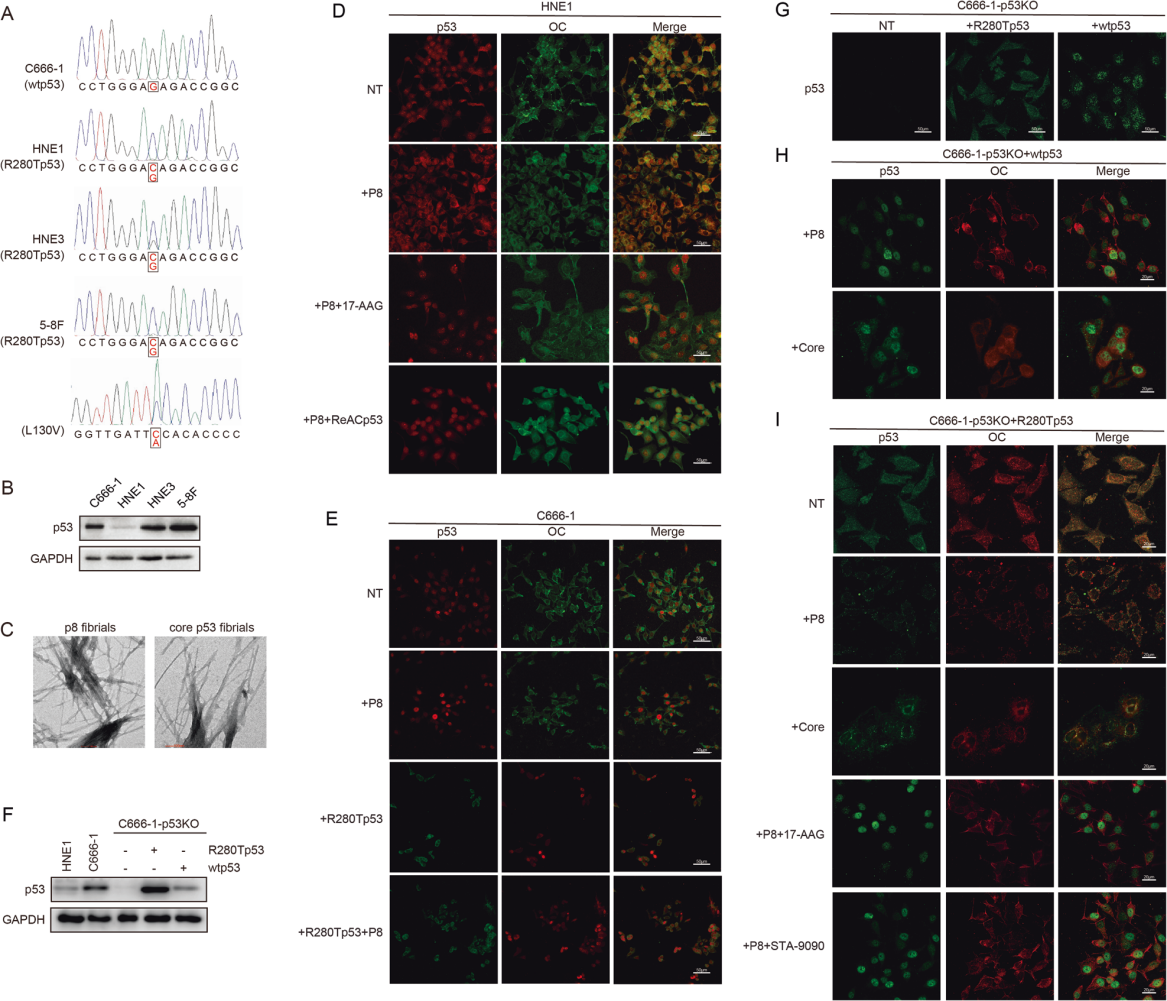
p53-R280T is extensive in NPC cells and promotes p53 amyloid formation and aggregation. **A** DNA sequencing showed the mutation status of the R280 site of p53 in C666-1, HNE1, HNE3 and 5–8 F cells. **B** Western blotting showed the expression of p53 in four NPC cell lines. GAPDH was used as the loading control. **C** Electron micrograph of P8 fibrils and core fibrils showing the fibrillar morphology of amyloids. Scale bars, 100 nm. **D** p53 aggregation in HNE1 cells with different treatments was detected by immunofluorescence staining. In the P8 fibrils treatment group, HNE1 cells were pretreated with 10 μM P8 fibrils for 24 h. In the P8 fibrils plus 17-AAG treatment group, HNE1 cells were treated with 50 nM 17-AAG for 24 h after P8 fibrils pretreatment. In the P8 fibrils plus ReACp53 treatment group, HNE1 cells were treated with 10 µM ReACp53 for 24 h after P8 fibrils pretreatment. NT indicates no treatment. Scale bars, 50 μm. **E** p53 aggregation in C666-1 cells with different treatments was detected by immunofluorescence staining. In the P8 fibrils treatment group, C666-1 cells were treated with 10 μM P8 fibrils for 24 h. In the R280Tp53 group, C666-1 cells were infected with lentivirus carrying p53-R280T. In the R280Tp53 plus P8 fibrils treatment group, C666-1 cells infected with p53-R280T lentivirus were treated with 10 μM P8 fibrils for 24 h. NT indicates no treatment. Scale bars, 50 μm. **F** Western blotting showed the expression of p53 in C666-1-p53KO and C666-1-p53KO cells infected with p53-R280T or wtp53. **G** Immunofluorescence showed p53 expression and location in C666-1-p53KO cells and C666-1-p53KO cells infected with p53-R280T or wtp53. Scale bars, 50 μm. **H** Immunofluorescence showed the states of p53 amyloid in C666-1-p53KO cells infected with wtp53 upon treatment with 10 μM P8 fibrils or core fibrils for 24 h. Scale bars, 20 μm. **I** Immunofluorescence showed the states of p53 amyloid in C666-1-p53KO cells infected with p53-R280T upon different treatments. In the P8 fibrils and core fibrils treatment groups, cells were pretreated with 10 μM P8 fibrils or core fibrils for 24 h. In the P8 fibrils plus 17-AAG or STA-9090 treatment groups, cells were treated with 50 nM 17-AAG or STA-9090 for 24 h after P8 fibrils pretreatment. Scale bars, 20 μm.

To explore whether R280T mutations were related to p53 aggregation in NPC, full-length TP53 was sequenced to check the mutation status in four NPC cell lines. The results showed R280T mutations in HNE1, HNE3, and 5-8F cells, specifically, a heterozygous change from AGA to ACA in codon 280 of p53. In addition to R280T, there is another L130V mutation of p53 in 5-8F cells. However, there was no discovery of any TP53 mutation in the C666-1 cell lines (Fig. 2A). Moreover, p53 expression was also evaluated in the four NPC cell lines, and the results showed that p53 was highly expressed in C666-1, HNE3 and 5-8F cells but expressed at low levels in HNE1 cells (Fig. 2B). To explore the association of the R280T mutant with p53 aggregate formation, HNE1 cells, which contain the R280T mutation, were chosen for further aggregation detection, and C666-1 cells with wild-type p53 (wtp53) were regarded as the negative control. ANS, which was demonstrated to promote p53 aggregation and the so-called peptide P8, was synthesized and processed into P8 fibrils in accordance with published studies [20]. In addition to P8 fibrils, the p53 core domain was also demonstrated to have potency in promoting p53 aggregation in vitro and in vivo [32–34]. To obtain amyloid fibrils of the p53 core domain (core fibrils), p53 core proteins (wtp53. AA: 92-292) were purified and incubated with chondroitin sulfate A (CSA) for several days. Both the P8 fibrils and core fibrils showed the fibrillar morphologies of amyloids under the electron micrographs (Fig. 2C).

To explore the primal p53 aggregation status in cells, immunofluorescence staining was performed in HNE1 and C666-1 cells. The results showed that some cytoplasmic p53 colocalized with OC signals in HNE1 cells, suggesting that there was a low amount of cytoplasmic p53 in the form of amyloid. When P8 fibrils were transfected into HNE1 cells for 24 h, p53 aggregation was stabilized as punctate structures in the cytoplasm and highly colocalized with the OC signals, suggesting that p53 amyloid formation was promoted by P8 fibrils in HNE1 cells. In addition, a classical Hsp90 inhibitor 17-AAG (tanespimycin), which can inhibit the function of Hsp90 and its interaction with p53 but does not change its expression, was introduced [35]. After treatment with 17-AAG for 24 h, p53 aggregation in HNE1 cells, which were pretreated with P8 fibrils, was decreased, and p53 protein recovered nuclear expression and seldom colocalized with OC signals. We also evaluated the potential effect of ReACp53, a cell-permeable peptide inhibitor for p53 aggregation, for R280T mutant aggregation [36]. Similar to 17-AAG, ReACp53 could dissociate aggregated p53 (Fig. 2D). Unlike HNE1 cells, wtp53 in C666-1 cells was constantly expressed in the nucleus even when transfected with P8 fibrils, and the colocalization of p53 with OC signals was negligible, indicating that p53 aggregation cannot be enhanced by P8 fibrils in cells expressing wtp53 (Fig. 2E). To determine the role of the R280T mutant in p53 aggregation, C666-1 cells were infected with lentivirus carrying the p53-R280T mutation (Supplementary Fig. 2A, B). After infection, the expression of exogenous p53-R280T and endogenous wtp53 in C666-1 cells was evaluated by fastNGS (Supplementary Fig. 2C). Specifically, the 202 bp amplified sequence containing the R280 site from cDNA of C666-1 was sequenced (Supplementary Fig. 2D), and the results showed that there was 43% stable exogenous p53-R280T of total p53 expression (Supplementary Table 1 and Supplementary Fig. 2E). As expected, after infection with p53-R280T lentivirus, C666-1 cells showed a similar potency of p53 aggregation to HNE1 cells, especially for those treated with P8 fibrils (Fig. 2E).

To further demonstrate the induction role of the R280T mutant in p53 aggregation formation, p53 was knocked out in C666-1 cell lines by using CRISPR/Cas9, and the expression of p53 was examined in C666-1 cells with p53 knockout (p53KO) or further infected with R280T or wtp53 by western blotting (Fig. 2F) and immunofluorescence (Fig. 2G). Immunofluorescence staining revealed that p53 was expressed in both the cytoplasm and nucleus of C666-1-p53KO cells infected with R280T, whereas it was expressed only in the nucleus of C666-1-p53KO cells infected with wtp53 (Fig. 2G). Furthermore, p53 aggregation was evaluated in C666-1-p53KO cells infected with R280T or wtp53 for treatment with P8 fibrils and core fibrils, separately. The results showed that similar to C666-1 cells, p53 aggregation could not be enhanced by exogenous fibrils in C666-1-p53KO cells infected with wtp53 (Fig. 2H). In contrast, when C666-1-p53KO cells were infected with R280T, mutant p53 aggregates were in the cytoplasm and highly colocalized with the OC signals after treatment with P8 fibrils or core fibrils. However, p53 aggregation was decreased by treatment with 17-AAG or another Hsp90 inhibitor, STA-9090, and p53 protein recovered nuclear expression (Fig. 2I).

### p53-R280T amyloid formation blocks its nuclear localization and transcriptional activation

**Fig. 3:**
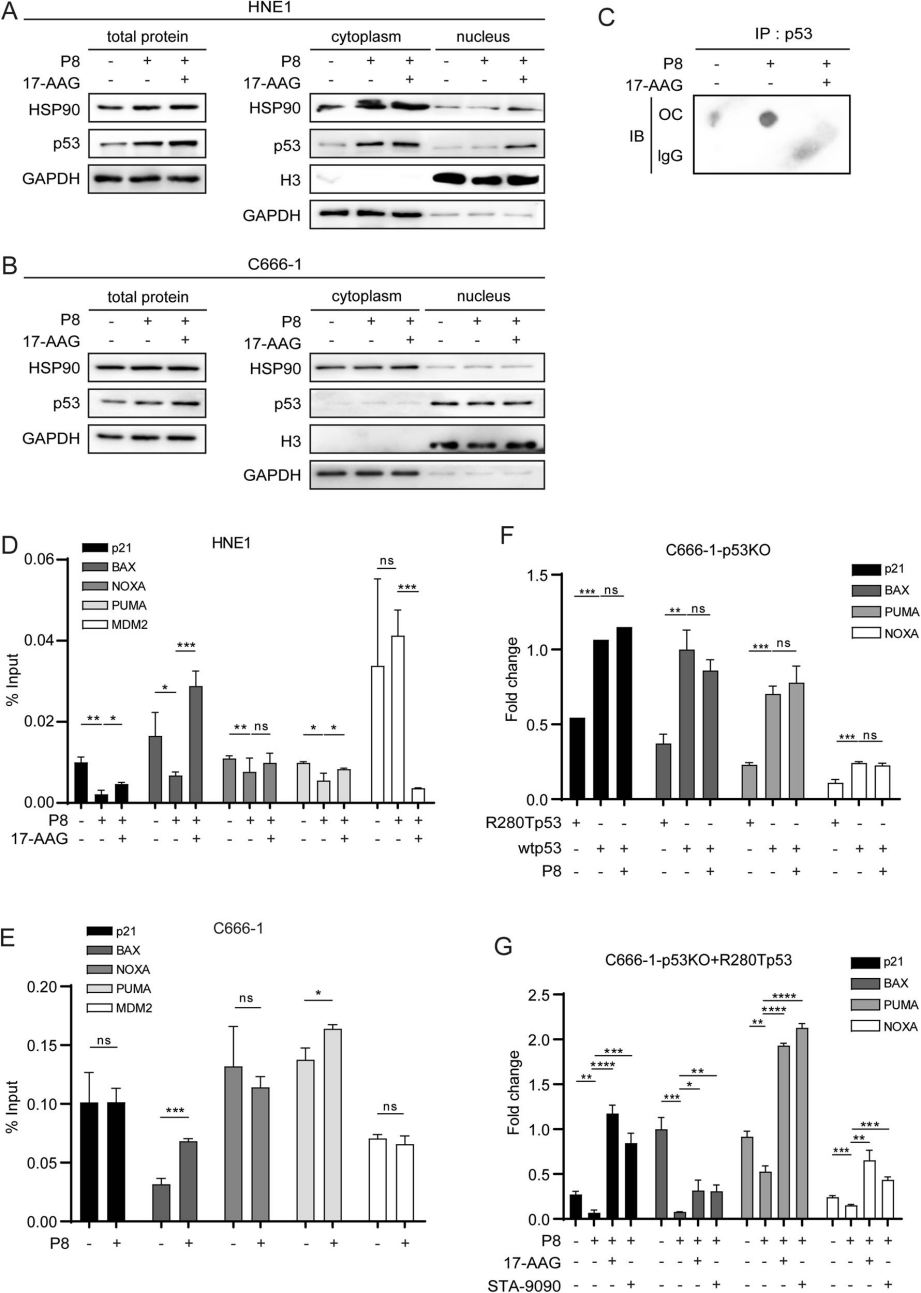
p53-R280T amyloid formation blocks its nuclear localization and transcriptional activity. **A** p53 and Hsp90 expression in the total protein, cytoplasm, and nucleus of HNE1 cells with different treatments was detected by western blotting. GAPDH was used as the loading control for total and cytoplasmic proteins, and H3 was the loading control for nucleic proteins. **B** p53 and Hsp90 expression in total protein, cytoplasm, and nucleus of C666-1 cells with different treatments was detected by western blotting. GAPDH was used as the loading control for total and cytoplasmic proteins, and H3 was the loading control for nucleic proteins. **C** p53 aggregation was detected by dot blot assay with OC antibody after p53 immunoprecipitation in HNE1 cells with different treatments. **D** p53 transcriptional activity for its target genes (p21, BAX, NOXA, PUMA and MDM2) in HNE1 cells with different treatments was detected by ChIP. **E** p53 transcriptional activity for its target genes (p21, BAX, NOXA, PUMA and MDM2) in C666-1 cells with different treatments was detected by ChIP. **F** qPCR analysis of the mRNA expression of target genes (p21, BAX, PUMA and NOXA) in C666-1-p53KO cells infected with R280Tp53 or wtp53 upon P8 fibrils treatment. **G** qPCR analysis of the mRNA expression of target genes (p21, BAX, PUMA and NOXA) in C666-1-p53KO cells infected with R280Tp53 upon P8 fibrils treatment or P8 plus 17-AAG or P8 plus STA-9090 treatments. Data are represented as mean ± SD. ns indicates no significance; *P < 0.05; **P < 0.01; ***P < 0.001; ****P < 0.0001; ChIP, chromatin immunoprecipitation.

To investigate the impact of p53 aggregation on p53 and Hsp90 expression or localization, nuclear and cytoplasmic proteins were extracted and evaluated by western blotting. We found that P8 fibrils could increase the expression of total p53 and Hsp90 proteins in HNE1 cells. Consistent with the immunofluorescence results, p53 expression in the cytoplasm of HNE1 cells was distinctly elevated with P8 fibrils treatment. In contrast, the expression in the nucleus was almost undetectable with or without P8 fibrils treatment. After 17-AAG treatment for 24 h, p53 protein expression in the nucleus was significantly increased (Fig. 3A). However, p53 was widely present in the nuclear extract of C666-1 cells and was not affected by P8 fibrils or 17-AAG (Fig. 3B). The immunoprecipitated p53-R280T proteins in HNE1 cells can also be detected by OC antibody, and the bands were even enhanced with the pretreatment of P8 fibrils and weakened by 17-AAG treatment (Fig. 3C). All these results suggested that P8 fibrils could enhance mutant p53-R280T protein aggregation, leading to its cytoplasmic retention but no impact on wtp53, and 17-AAG could recover the native status and nuclear expression of p53-R280T.

To further examine the impact of p53 aggregation on its DNA-binding activity, chromatin immunoprecipitation was performed. The results showed an obvious transcriptional reduction in p53 downstream key target genes (p21, BAX, NOXA, and PUMA) with P8 fibrils treatment, and 17-AAG could recover their transcription. MDM2, as the mediator of p53 degradation, was inversely regulated by 17-AAG (Fig. 3D). Nevertheless, the transcription of the downstream target genes of p53 mentioned above was not affected by P8 fibrils in C666-1 cells, and BAX and PUMA even increased with P8 fibrils treatment (Fig. 3E).

To further evaluate the changes in transcriptional regulation induced by p53 aggregation, we detected the mRNA expression of four target genes regulated by p53 in C666-1-p53KO cells infected with R280T or wtp53 upon different treatments. The qPCR results showed that the expression of p21, BAX, PUMA and NOXA was downregulated in C666-1-p53KO cells infected with R280T compared to C666-1-p53KO cells infected with wtp53, and their expression in C666-1-p53KO cells infected with wtp53 was still not changed with P8 fibrils treatment (Fig. 3F). In contrast, significantly decreased expression of the four target genes was observed in C666-1-p53KO cells infected with R280T upon p53 amyloid formation induced by P8 fibrils. Moreover, the expression of the four target genes was distinctly elevated in C666-1-p53KO cells infected with R280T upon treatment with the two Hsp90 inhibitors due to their possible dissociation of p53 aggregation (Fig. 3G).

### p53-R280T amyloid formation causes p53 loss of function in vitro

**Fig. 4:**
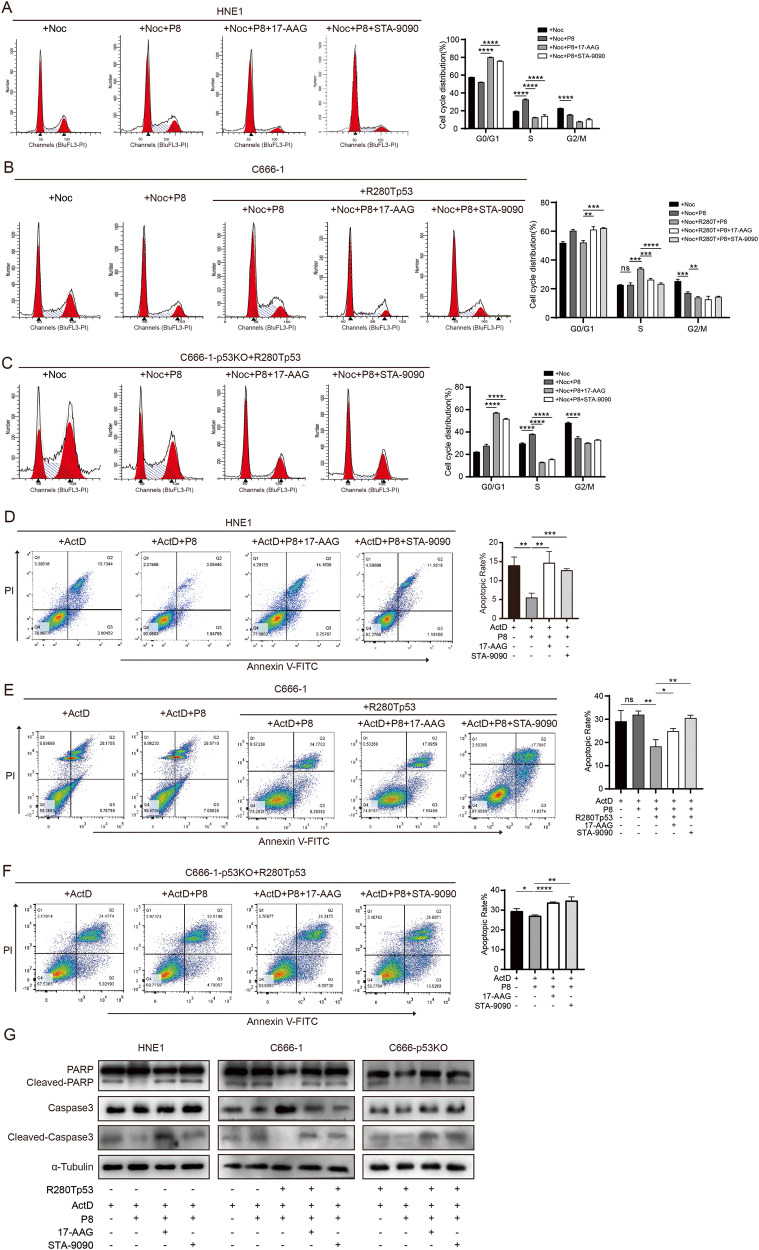
p53-R280T amyloid formation causes p53 loss of function in vitro. **A** Flow cytometry showed the effect of p53 aggregation on cell cycle progression in HNE1 cells. The right panel indicates the statistical analysis. **B** Flow cytometric analysis showed the cell cycle results of C666-1 cells with different treatments. The right panel indicates the statistical analysis. **C** Flow cytometry showed the effect of p53 aggregation on cell cycle progression in C666-1-p53KO cells infected with R280Tp53. The right panel indicates the statistical analysis. **D** Flow cytometry showed the effect of p53 aggregation on cell apoptosis in HNE1 cells. The right panel shows the statistical analysis. **E** Flow cytometric analysis showed the cell apoptosis results of C666-1 cells with different treatments. The right panel shows the statistical analysis. **F** Flow cytometry showed cell apoptosis in C666-1-p53KO cells infected with R280Tp53 upon different treatments. The right panel shows the statistical analysis. **G** Western blotting showed the expression of caspase 3, cleaved caspase 3, PARP and cleaved PARP in HNE1, C666-1 and C666-1-p53KO cells with different treatments. α-Tubulin was used as the loading control. Data are represented as mean ± SD. ns indicates no significance; *P < 0.05; **P < 0.01; ***P < 0.001; ****P < 0.0001.

The most common p53 inactivation in tumor cells is the inability to activate cell cycle arrest and induce apoptosis. Therefore, flow cytometric analysis was performed to evaluate the effect of p53 amyloid aggregation on the NPC cell cycle and apoptosis. The microtubule destabilizing drug nocodazole (Noc) and the DNA-damaging agent actinomycin D (ActD) were used as inducers during the cell cycle and apoptosis detection, respectively. The results showed that there was a significant accelerated S phase progression of HNE1 cells by treatment with P8 fibrils, whereas 17-AAG and STA-9090 could hinder the accelerated cell cycle (Fig. 4A). It seems that P8 fibrils could not accelerate the cell cycle for C666-1 cells. However, when infected with R280T, S phase progression was accelerated in both C666-1 and C666-1-p53KO cells upon P8 fibrils treatment, and 17-AAG or STA-9090 could hamper the accelerated cell cycle and arrest cells at G0/G1 phase (Fig. 4B, C). Similarly, flow cytometric analysis also revealed a significant reduction in cell apoptosis in HNE1 cells, as well as C666-1 and C666-1-p53KO cells infected with R280T, upon P8 fibrils treatment. In addition, 17-AAG or STA-9090 treatment reversed the decreased cell apoptosis induced by P8 fibrils (Fig. 4D, E and F). Furthermore, cleaved caspase 3 and cleaved PARP were also examined by western blotting to determine the effect of p53 aggregation formation on cell apoptosis. The results showed that cleaved caspase 3 and cleaved PARP in HNE1 cells, as well as C666-1 and C666-1-p53KO cells infected with R280T, were all reduced upon P8 fibrils treatment and recovered upon 17-AAG or STA-9090 treatment (Fig. 4G).

### p53-R280T amyloid formation induces tumorigenesis, and 17-AAG exhibits therapeutic effects in vivo

**Fig. 5:**
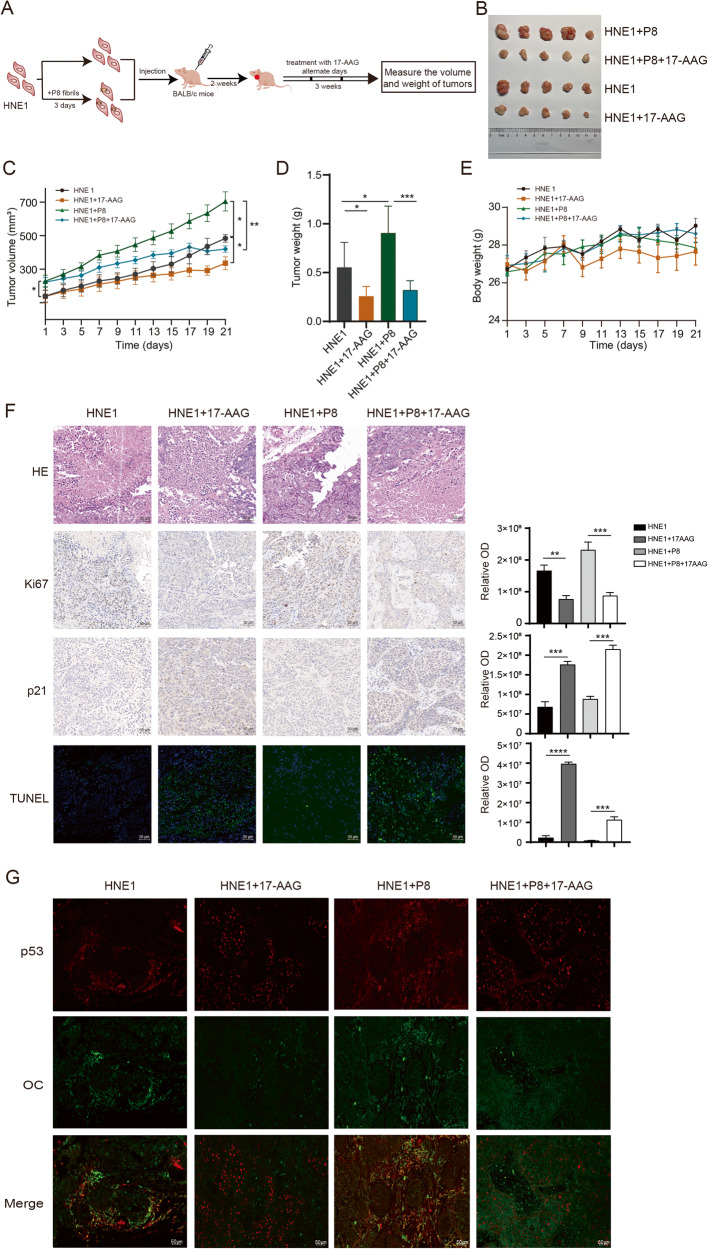
p53-R280T amyloid formation induces NPC tumorigenesis in vivo, and 17-AAG exerts a therapeutic effect. **A** Flow chart of the NPC xenograft experiments. **B** Images of excised tumors from mice in each group with different treatments. **C** Tumor growth curves of mice in each group with different treatments. **D** Tumor weight of mice in each group with different treatments at the end of the experiment. **E** The change in body weight of mice in each group with different treatments during the experimental process. **F** Representative images of HE staining, IHC staining for Ki67 and p21, and immunofluorescence staining of TUNEL in tissue sections of tumors from each group with different treatments. Scale bars, 50 μm. The right panel shows the statistical analysis for the quantitation of Ki67, p21 and TUNEL. **G** Immunofluorescence staining with p53 and OC antibodies showed the p53 aggregation status in tumors from each group with different treatments. Red represents p53 antibody-specific staining, and green represents OC antibody (specific to amyloids) staining. Scale bars, 50 μm. Data are represented as mean ± SD. *P < 0.05; **P < 0.01; ***P < 0.001; ****P < 0.0001. HE hematoxylin and eosin, IHC immunohistochemistry.

To confirm the impact of p53 amyloid formation on tumorigenesis in vivo, xenograft models of NPC were constructed by subcutaneous injection of HNE1 cells or HNE1 cells pretreated with P8 fibrils into nude mice. The xenograft experiment flow is shown in Fig. 5A. During the tumor growth process, the group injected with HNE1 cells pretreated with P8 fibrils showed a faster tumor growth rate, and the tumor volume was also even greater at the end of the experiment. 17-AAG remarkably suppressed tumor growth in both groups but was more effective in the HNE1 with P8 fibrils pretreatment group (Fig. 5B, C, D). Meanwhile, 17-AAG had no effect on the body weight of the mice (Fig. 5E).

The isolated tumors from the four groups were fixed, embedded, and sectioned. HE staining and IHC showed that both 17-AAG treatment groups displayed weaker staining of Ki67 and enhanced staining for p21 and TUNEL compared with the HNE1 group or HNE1 with P8 fibrils pretreatment group. The quantitative analysis of Ki67, p21 and TUNEL staining also suggested that 17-AAG could significantly inhibit NPC growth and promote apoptosis in vivo (Fig. 5F). Moreover, immunofluorescence was conducted to detect p53 aggregation in each group using p53 antibody and OC antibody. The results showed that aggregates were enhanced in the HNE1 with P8 fibrils pretreatment group. 17-AAG significantly reduced the colocalization of p53 with OC signals and stabilized p53 expression in the nucleus (Fig. 5G). All these results suggested that p53-R280T amyloid formation lead to greater tumorigenic properties and that 17-AAG showed significant therapeutic efficacy by suppressing p53 aggregation in vivo.

### Transcriptome alteration of NPC cells caused by p53 aggregation induced by the R280T mutation

**Fig. 6:**
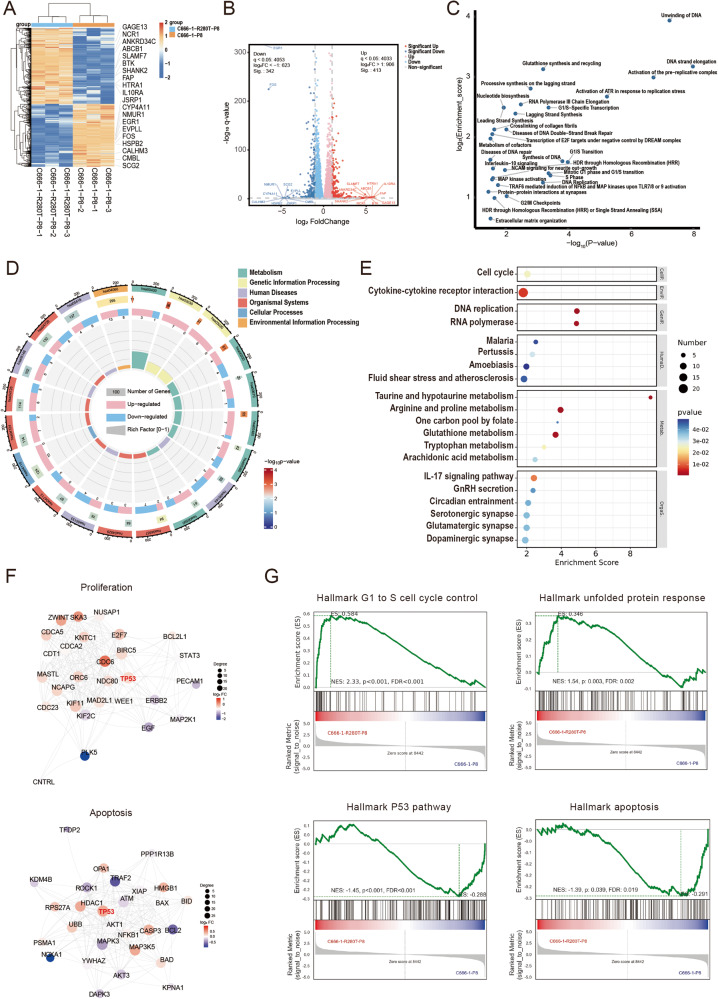
Transcriptome analysis of the impact of p53-R280T amyloid formation in NPC cells. **A** Heatmap of cluster analysis for differentially expressed genes between C666-1 cells infected with R280T and C666-1 upon treatment with P8 fibrils. Orange indicates relatively high gene expression, and blue indicates relatively low gene expression. **B** Volcano map of differentially expressed genes between C666-1-R280T and C666-1 cells upon treatment with P8 fibrils. Red and blue represent the significantly upregulated and downregulated genes, respectively. Gray represents nonsignificant differentially expressed genes. **C** Plot of the *P* value and enrichment ratio of significantly altered signaling pathways between C666-1-R280T and C666-1 cells upon treatment with P8 fibrils. **D** Circle plot of GO analysis of the differentially expressed genes between C666-1-R280T and C666-1 cells upon treatment with P8 fibrils. **E** KEGG enrichment analysis for differentially expressed genes between C666-1-R280T and C666-1 cells upon treatment with P8 fibrils. **F** PPI network analysis of dysregulated p53 target proteins altered upon p53-R280T amyloid formation. The red and blue bubbles represent the upregulated and downregulated p53 target proteins, respectively. The size of the dots indicates the number of interacting proteins. **G** GSEA of the cell cycle, unfolded protein response, p53 pathway, and apoptosis in C666-1-R280T cells compared with C666-1 cells upon treatment with P8 fibrils.

To explore the global effect on signaling pathways and the underlying mechanism of p53-R280T amyloid formation in NPC, transcriptome sequencing was performed for C666-1, C666-1 pretreated with P8 fibrils (C666-1-P8) and C666-1 infected with R280T plus P8 fibrils treatment (C666-1-R280T-P8). Among the three groups, C666-1-P8 vs C666-1 or C666-1-R280T-P8 vs C666-1-P8 were compared. Filtering with *p* < 0.05 and Log_2_FC > 1, DEG analysis identified 755 mRNAs in C666-1-R280T-P8 vs C666-1-P8, among which 413 were upregulated (55%) and 342 were downregulated (45%) (Fig. 6A, B), while 2255 differentially expressed mRNAs were identified in C666-1-P8 vs C666-1, among which 1337 were upregulated (59%) and 918 were downregulated (40%) (Supplementary Fig. 3A, B).

GO and KEGG analyses revealed that upon P8 fibrils treatment, the upregulation of numerous mRNAs or signaling pathways involved in DNA replication, synthesis and metabolic processing was higher in C666-1 cells infected with R280T than in C666-1 cells carrying wtp53, which provided a tumor growth advantage (Fig. 6C, D, E). However, similar results were not observed when comparing C666-1 cells treated with P8 fibrils to C666-1 cells, indicating that P8 fibrils could not promote p53 aggregation in unmutated NPC cells (Supplementary Fig. 3C). The differentially expressed genes for p53-centered genes were analyzed to establish the network of p53-regulating target genes. The results showed that a large number of genes involved in cell cycle regulation and proliferation signaling pathways (CDC6, other CDCs) were upregulated, and many apoptosis-related genes (BCL2, NOXA) were significantly downregulated in C666-1 cells infected with R280T upon P8 fibrils treatment (Fig. 6F). GSEA revealed that genes associated with the cell cycle and DNA replication along with the main related signaling pathways were significantly upregulated, and genes involved in the p53 pathway and apoptotic pathways were downregulated in C666-1 cells infected with R280T upon P8 fibrils treatment, which might be the leading causes of p53 amyloid formation and accumulation facilitating NPC progression (Fig. 6G). In addition, the expression of Hsp70 and Hsp90 chaperones was significantly elevated, and the unfolded protein response (UPR) was upregulated in C666-1 cells infected with R280T upon P8 fibrils treatment, which might in turn promote p53 amyloid formation (Fig. 6G). In contrast, compared with C666-1 cells, the cell cycle, DNA replication, DNA repair, and UPR pathways were not altered distinctly or were even downregulated in C666-1 cells treated with P8 fibrils, suggesting that p8 fibrils had no aggregation effect on wtp53 in C666-1 cells (Supplementary Fig. 3D, E).

### p53 loss of function is linked to p53 amyloid aggregates induced by the R280T mutant and forms positive feedback with Hsp90 in NPC cells

**Fig. 7:**
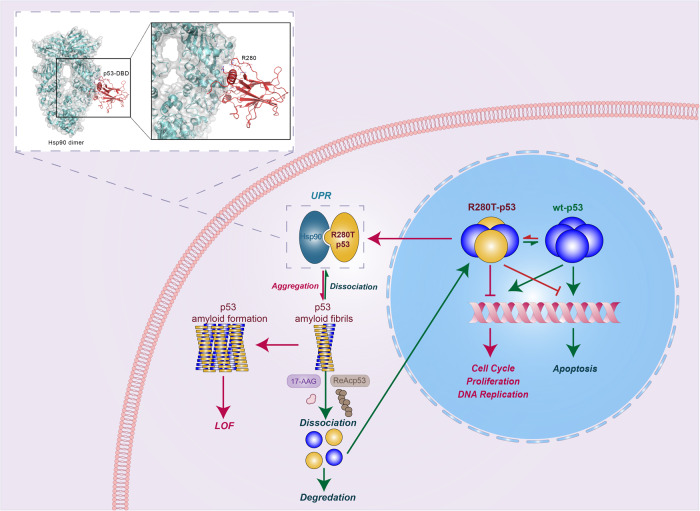
Model of p53 loss of function induced by p53-R280T amyloid aggregates in NPC cells. A docking model of the Hsp90 dimer in complex with p53-DBD is shown as a cartoon at the top of the left corner. Residues in the protein‒protein interface are indicated and shown in lines. p53-R280 is shown in stick representation. The Hsp90 dimer is colored cyan. p53-DBD is colored brick-red. p53 functions as a transcription factor in tetrameric form. Wild-type p53 is indicated in dark orchid, and R280T-p53 is indicated in yellow. Red arrows indicate LoF affected by amyloid formation of p53-R280T, and green arrows indicate the normal situation or recovery of p53 function.

To better present the association of p53 and Hsp90 in the p53 aggregation process, the Hsp90-p53 docking model was built by the HADDOCK web server as previously described [29], and the p53 aggregation pathway was constructed according to our results (Fig. 7). The Hsp90-p53 docking model showed that the R280 site is at the interaction face of p53 and Hsp90; therefore, the R280T mutation would destroy the interaction and lead to p53 misfolding, amyloid formation in the cytoplasm, and even aggregation with native p53. The mechanistic model showed that aggregated p53 cannot enter the nucleus to bind its target genes and initiate their transcription, leading to the loss of its tumor suppressive function, such as cell cycle arrest, DNA replication inhibition and apoptosis promotion. Meanwhile, the chaperones Hsp90 and UPR, which play crucial roles in p53 folding, were highly upregulated in p53-aggregated NPC cells. The upregulated Hsp90 in turn further assisted in p53 aggregation, ultimately forming positive feedback. In addition, the Hsp90 inhibitor 17-AAG and the aggregation inhibitor ReACp53 could dissociate the amyloid aggregation of the R280T mutant p53 protein complex, and the dissociated p53 could restore its expression in the nucleus and exert its suppressive function or was degraded through ubiquitination.

## Discussion

Prion diseases, caused by the misfolding and aggregation of prion proteins [37], are also known as protein misfolding diseases and primarily affect the nervous system [38]. In recent years, p53 aggregation has been found in various tumors, and the inactivation of p53 suppressor function caused by aggregation is a contributing factor in tumor pathogenesis. Unlike other tumor types, NPC exhibits high levels of p53 expression but a low mutation frequency, and the underlying mechanism responsible for p53 LoF in NPC remains unclear. Thus, we investigated whether p53 aggregation exists in NPC and examined it in an NPC tissue microarray. The results showed that up to 83.3% of 90 NPC samples were p53 positive, and 26.6% of the p53-positive samples were colocalized with OC signals, indicating that p53 aggregation is ubiquitous in NPC. The dot blot assays for TAF of NPC confirmed that the degree of aggregation was more significant with increased p53 expression.

Although few reports have focused on p53 mutations in NPC, the R280T mutation, not a hotspot mutation, has been reported to occur in approximately 10% of NPC cases [9]. Regrettably, we were not able to obtain the p53 mutation rates of the tissue microarray used in this study because there were no additional NPC tissues for sequencing. However, three out of four NPC cell lines were identified to carry R280T heterozygous mutations in this study. Other studies also identified R280T mutations in five distinct NPC cell lines (CNE1, CNE2, TW06, TW01, and HONE1) [7, 9, 39]. The high incidence of the R280T mutant in NPC cells was unexpected and exceeded the 10% probability reported in NPC patients. Therefore, 26.6% of the p53 aggregation probability detected in the NPC tissue microarray in this study may have other unknown reasons, apart from the R280T mutation [19]. For instance, abnormal expression of isoforms may induce p53 aggregation [40]. Other factors may also contribute to p53 amyloid aggregation, such as RNA, environment, Zn^2+^ concentration, pH, temperature, and chaperone abnormality [41–44]. Moreover, p53 aggregation may not be attributed to a singular cause but rather can result from a combination of various factors.

Hsp90, a well-known chaperone, is frequently overexpressed in various tumors and plays a crucial role in p53 folding [45]. p53 is one of the substrates for Hsp90 [46]. Under normal physiological conditions, wtp53 transiently interacts with Hsp90 to maintain its activity and undergoes degradation through ubiquitination-mediated processes [47]. However, in tumor cells, the disrupted interaction between p53 and Hsp90 could inhibit p53 degradation, leading to the accumulation and aggregation of p53 proteins, ultimately resulting in the loss of their tumor suppressive function [47, 48]. The R280 site is located on the section of the DNA interaction surface of p53 α-Helix2, which is one of the interaction sites of Hsp90 [31]. Additionally, arginine is known to prevent protein aggregation. Therefore, the R280T mutation may affect the interaction of p53 with Hsp90 and promote p53 aggregation, as validated in our study. In addition, significantly elevated Hsp70 and Hsp90 chaperones, along with upregulated UPR, were observed in the transcriptome sequencing for p53-R280T aggregated NPC cells, suggesting that cells may activate defense mechanisms against misfolded protein aggregation and tumor promotion. The expression of Hsp90 was also found to be elevated along with p53-R280T aggregation in NPC cells. However, upregulated Hsp90, in turn, promoted p53 aggregation instead of inhibiting it, forming a positive feedback loop. This observation aligns with the findings of Navalkar et al., who observed that Hsp90 responded to the upregulation of aggregated p53 in cells [49]. Xu et al. also reported that the overexpression of aggregated p53-R175H significantly upregulated Hsp70 and Hsp90 [21]. The upregulation of the cell cycle, DNA replication, and metabolic pathways, along with increased oncogene expression, confirmed that p53-R280T aggregation obtains precancerous properties by inactivating p53 and upregulating Hsp90. MDM2, a negative regulator of p53, could inhibit the stability and transcriptional activity of p53 by ubiquitination-mediated degradation. MDM2 is one of the target genes of p53, and its expression level is regulated by p53 [50, 51]. The ChIP results showed that MDM2 expression was significantly downregulated after the dissociation of aggregated p53 by 17-AAG, which may be one of the reasons for the increased stability of p53 and the restoration of its inhibitory function.

More than 200 different single-site mutations have been reported for TP53. Some of them were classified into structural mutants and contact mutants. Structural mutations, such as R175, R248, R249, and M237, are thought to disrupt the stability of the DBD and increase the tendency to aggregate [52]. Contact mutants such as R280 and R273 directly interact with DNA. These sites are essential for DNA binding, and their mutation may impair the transcriptional activity of wtp53 [53]. It was believed that the conformation of p53 may not be significantly affected by contact mutants [21]. However, p53 aggregation in tissues containing contact mutants has been observed in numerous studies [15, 54–56]. In our study, we proposed, for the first time, the aggregation tendency of the R280 mutation, thus expanding the research on p53 aggregation in tumors. ReACp53, as a specialized anti-aggregation inhibitor [36], could dissociate the p53 aggregation induced by the R280T mutation, suggesting its promising therapeutic potential in p53-aggregated NPC. Additionally, Hsp90 inhibitors, which show promise in tumor therapy, were also found to have a more pronounced effect on tumors containing p53 aggregates. This finding may pave the way for the development of a new field for Hsp90 inhibitor therapy. Overall, our studies provide valuable insights into the underlying mechanism and potential therapeutic intervention for NPC.

## Materials and methods

### Cell culture

The human NPC cell lines HNE1, HNE3 and 5-8F were preserved in the NHC Key Laboratory of Cancer Proteomics of Xiangya Hospital, Central South University. C666-1 cells were purchased from Meisen Chinese Tissue Culture Collections, Zhejiang, China. The C666-1-p53KO cells were kindly gifted by Dr. Wei Huang. All cells were cultured in RPMI-1640 medium (Gibco, Grand Island, USA) supplemented with 10% FBS (Gibco), 10 U/ml penicillin, and 100 μg/ml streptomycin (Gibco). Cells were grown at 37 °C and 5% CO_2_ in a humidified incubator. No mycoplasma contamination was detected.

### Immunofluorescence of cells and tissues

The cultured cells were treated with 10 μM P8 fibrils or core fibrils, P8 fibrils plus 50 nM 17-AAG (Selleck Chemicals, Houston, USA) or STA-9090 (MedChem Express, Monmouth Junction, USA), and P8 fibrils plus 10 μM ReACp53 for 24 h. NPC and breast carcinoma tissue sections were obtained from the Department of Pathology, Xiangya Hospital, Central South University. Both cells and tissue slices were fixed with 4% paraformaldehyde for 20 min, and the sections or cells were permeabilized with 0.2% Triton X-100 for 10 min. PBST containing 2% BSA was used to block nonspecific antigenic sites. Then, the coverslips were incubated with primary antibodies in a humidified chamber overnight at 4 °C. The antibodies used were as follows: p53 antibody (#ab1101, Abcam) (1:200 dilution), OC antibody (beta Amyloid Polyclonal Antibody, #600-401-253, Rockland) (1:500 dilution), and A11 antibody (Amyloid Oligomers Antibody, #200-401-E88, Rockland) (1:500 dilution). After washing with PBS three times, the coverslips were incubated with FITC-conjugated goat anti-mouse (#A22110, Abbkine) or goat anti-rabbit Dylight-594 (#A23420, Abbkine) secondary antibodies in a humidified chamber for 2 h at room temperature. Confocal microscopy (Olympus FluoView 1000 laser scanning, Tokyo, Japan) was used to obtain confocal microscopy images.

### Human tissue amyloid fibril extraction

Human NPC or NNET tissues used for amyloid fibril extraction were collected from the Department of Otolaryngology Head and Neck Surgery, Xiangya Hospital, Central South University. The isolation of amyloid fibrils was conducted according to published protocols [57]. Human tissues (250–500 mg) were minced and homogenized in 500 μL of 0.15 M NaCl (250 mg tissue) for 15 min. Then, at 4 °C, the tissues were centrifuged at 8,600×g for an hour. After rehomogenization with 0.15 M NaCl for 15 min, the pellet was centrifuged at 8600×g for 1 h at 4 °C. Afterward, homogenization of the pellet was performed in 500 M Tris-HCl, 3 M NaN_3_, and 0.01 M CaCl_2_, pH 7.5. A ratio of 1:100 collagenase type I powder (Himedia, Mumbai, India) was added to the tissue overnight at 37 °C. The mixture was centrifuged at 74,000×g for 1 h on an ultracentrifuge (Beckmann Coulter, Miami, USA) at 4 °C, followed by homogenization in 300 μL of 0.15 M NaCl and centrifugation at 74,000×g for another hour. The procedure was repeated several times until the absorbance of the supernatant was less than 0.3. Following homogenization in 200 µL of distilled water, the pellet was centrifuged at 4 °C and 38,000×g for 1 h. A repeat homogenization of pellets was performed until clear supernatants were collected. Next, the amyloid fibrils were precipitated using 0.15 M NaCl was added to a mixed supernatant. The mixture was centrifuged at 74,000×g and 4 °C for another hour. The final pellet with amyloid fibrils was stored at 4 °C and suspended in PBS prior to use.

### Dot blot

A 4 μL sample of extracted amyloid fibril was spotted onto nitrocellulose membranes (Merck Millipore, Billerica, USA) and air-dried. Then, the membranes were blocked with 5% nonfat milk at room temperature for an hour and washed in TBST, followed by incubation with different primary antibodies for 1 h at room temperature. The primary antibodies used in this study were as follows: p53 (1:200 dilution), OC (1:500 dilution), and A11 (1:500 dilution). After that, the blots were washed with TBST three times for 10 min and then incubated with a secondary antibody conjugated with HRP for another hour at room temperature. Visualization of the signal was performed using an enhanced chemiluminescence detection kit (ECL) (Vazyme, Nanjing, China).

### Detection of mutation types in NPC cell lines

Genomic DNA was extracted from C666-1, HNE1, HNE3, and 5-8F cell lines by a DNA extraction kit (Tiangen, Beijing, China), and then DNA was analyzed by Sanger sequencing to detect TP53 mutations. PCR amplification on both strands and DNA sequencing confirmed the mutation in the samples. Exon 8 of the TP53 gene was amplified by oligonucleotide primers. The primers used are listed in Supplementary Table 2. Sequencing was performed at Sangon Biotech in Shanghai, China.

### P8 fibrils preparation and transmission electron microscopy

P8 fibrils were prepared following the published protocol [20]. A concentration of 1 mM synthesized peptide P8 (NH2-PILTIITL-COOH) was dissolved in 1.5 mL of 5% D-mannitol (Solarbio, Beijing, China) and 0.01% sodium azide (pH 5.5). P8 suspensions were sonicated with bath sonicators to make clear solutions. The P8 peptide solutions were incubated for more than 8 h at a speed of 50 rpm on a rotating mixture at 37 °C. Then, the sample was observed by transmission electron microscopy (TEM). Spotting of P8 fibrils was performed on copper-coated Formvar grids, followed by washing with Milli-Q water and staining for 20 min with 0.1% uranyl formate solution. Before use, a 0.22 mm sterile syringe filter was used to filter the uranium formate solution (Merck Millipore). TEM images were taken at 120 kV on an electron microscope (FeiTecnai12 D312, Waltham, USA).

### Purification of the wtp53 core domain and core fibrils preparation

The human p53 core domain (amino acids 92-292) was cloned and inserted into the pGEx-6P1 expression vector (GE healthcare, Little Chalfont, UK) with a PreScission site between the GST tag and p53. The recombinant proteins were expressed in *E. coli* BL21 (DE3) cells (TransGen Biotech, Beijing, China). For purification, the cells were resuspended with a high-pressure homogenizer in lysis buffer (20 mM sodium citrate pH 6.3, 500 mM NaCl, 10 μM Zn^2+^ and 5 mM β-mercaptoethanol). GST-p53-DBD protein was purified by glutathione sepharose (GE Healthcare). The GST tag was removed by incubation with PreScission protease at 4 °C overnight. The product was further purified by cation-exchange chromatography (Mono S 5/50 GL, GE Healthcare) and Superdex 75 10/300 GL. The final protein was concentrated to approximately 10 mg/ml.

The purified wtp53 core domain was incubated in sodium phosphate buffer (50 mM), 0.3 M NaCl, pH 7.4 in the presence of an equimolar concentration of chondroitin sulfate A (CSA) (Aladdin, Shanghai, China). The mixture was rotated at 37 °C with slight agitation (50 rpm) for six days. The precipitate was resuspended in sterile PBS to obtain the core fibrils. TEM images of the core fibrils were also recorded.

### Molecular cloning, virus production, and cell infection

The pCDH-CMV plasmid expressing p53-R280T and wtp53 cDNA were generated by routine molecular cloning techniques. The R280T mutation was detected using Sanger sequencing. The plasmids of p53-R280T or wtp53 were transfected into HEK293T cells using Lipofectamine® 2000 (Invitrogen, Carlsbad, USA) and packaged into virus following standard protocols. Virus was collected and pooled from the supernatant of the transfected HEK293T cells. Cell infection was performed following the manufacturers’ instructions. Before infection, cells were grown in 6-well plates. Infection was carried out by adding p53-R280T or wtp53 lentivirus to 1 mL of Opti-MEM medium (Invitrogen) and 1 µl of polybrene reagent to the cells, and the cells were incubated for at least 8 h.

### Western blot

Cells were lysed with RIPA buffer (Beyotime Institute of Biotechnology, Shanghai, China) for protein extraction. Following the manufacturer’s instructions, the protein extracts were isolated by the Nuclear and Cytoplasmic Protein Extraction kit (#P0028, Beyotime Institute of Biotechnology). Proteins were separated by SDS‒PAGE and then blotted onto PVDF membranes (Merck Millipore). Blots were incubated with the following antibodies overnight at 4 °C: p53 (#ab1101, Abcam) (1:1000 dilution), HSP90 (rabbit polyclonal antibody, #13171-1-AP, Proteintech) (1:1000 dilution), PARP (rabbit polyclonal antibody, #13371-1-AP, Proteintech) (1:1000 dilution), Caspase3 (rabbit polyclonal antibody, #9664, CST) (1:1000 dilution), α-Tubulin (mouse monoclonal antibody, #ABL1080, Abbkine) (1:5000 dilution), histone-H3 (rabbit polyclonal antibody, #17168-1-AP, Proteintech) (1:1000 dilution) and GAPDH (rabbit polyclonal antibody, #10494-1-AP, Proteintech) (1:5000 dilution). Then, the membranes were incubated with HRP-conjugated secondary antibody for an hour at room temperature. Visualization of the blots was achieved using an ECL kit (Vazyme, Nanjing, China).

### Immunoprecipitation of p53 from cells

HNE1 cells were cultured with 10 μM P8 fibrils for 24 h or 50 nM 17-AAG for another 24 h. Immunoprecipitation was performed in accordance with the manufacturer’s instructions for Pierce™ (#26146, Thermo Fisher Scientific, Waltham, USA). IP lysis buffer was used to lyse cells with constant stirring for 30 min at 4 °C, and 12,000 rpm centrifugation was performed for 20 min at 4 °C. p53 antibody (2 μg) was added to the supernatant and incubated overnight at 4 °C under rotation. After adding 100 μL of Sepharose A/G Plus, incubation was carried out at 4 °C for 4 h with rotary agitation followed by centrifugation at 3000 rpm for 20 s. For protein elution, 150 µL of 0.2 M glycine buffer, pH 2.6, was incubated for 10 min at 4 °C with frequent agitation and centrifugation for 2 min at 3000 rpm. In an equal volume of Tris HCl, pH 8.0, the eluates were pooled and neutralized.

### p53 chromatin immunoprecipitation (ChIP)

To conduct ChIP studies, 2.5 × 10^5^ HNE1 or C666-1 cells were treated with 10 μM P8 fibrils or 10 μM P8 fibrils plus 50 nM 17-AAG for 24 h. ChIP was performed by following the manufacturer’s instructions for the SimpleChIP® Enzymatic Chromatin IP Kit (#9003, Cell Signaling Technology, Danvers, USA). qPCR was performed by using a ChamQ Universal SYBR qPCR Master Mix Kit (#Q711, Vazyme, Nanjing, China) on a LightCycler® 480 (Roche, Basel, Switzerland). The primers used are listed in Supplementary Table 2. qPCR was performed in triplicate for all ChIP experiments, and error bars depicting standard deviations are shown for all values. The input values were calculated using the following equation: ΔCT = CT(ChIP)-[CT(Input)-LogE(Input dilution factor)], % Input=E-ΔCT (E: specific primer efficiency value).

### RNA isolation and qRT‒PCR

Cells were cultured and treated with 10 μM P8 fibrils, followed by treatment with an additional 50 nM 17-AAG or STA9090. Untreated cells were used as corresponding controls. RNA was isolated from cells by TRIzol (Invitrogen) following the manufacturer’s instructions. The RNA concentrations were determined by a NanoDrop 2000 spectrophotometer (Thermo Fisher Scientific). The HiScript® III RT SuperMix for qPCR Kit (Q225, Vazyme, Nanjing, China) was used for reverse transcription. Then, the relative mRNA levels were detected using the ChamQ Universal SYBR qPCR Master Mix Kit (#Q711, Vazyme, Nanjing, China). The primers used are listed in Supplementary Table 2.

### Flow cytometry analysis of the cell cycle and apoptosis

For cell cycle detection, cells were treated with 10 μM P8 fibrils or P8 fibrils plus 50 nM 17-AAG or STA-9090 for 24 h, followed by treatment with Noc (5 nM) (MedChem Express) for another 24 h. The cells were washed with PBS and fixed with 75% ethanol at 4 °C. The fixed cells were kept at 4 °C for more than 18 h. Before staining, the cells were washed two times with PBS. Then, 200 μL PI/RNase Staining Buffers (BD Biosciences, San Jose, USA) were added to the cells and incubated for 15 min for DNA staining. Then, the cells were detected by flow cytometry. For cell apoptosis detection, cells were cultured and treated with 10 μM P8 fibrils or P8 fibrils plus 50 nM 17-AAG or STA-9090 for 24 h, followed by treatment with ActD (2 μg/mL) (MedChem Express) for another 24 h. Cells were stained with an Annexin V-Alexa Fluor 647/PI kit (#FXP023, 4 A Biotech, Suzhou, China) for apoptosis according to the manufacturer’s instructions. Apoptotic cells were quantified with a DxP Athena™ flow cytometer (Cytek Biosciences, CA, USA) and then analyzed by FlowJo V10 software. Triplicates of all assays were performed.

### Mouse xenograft studies

Male BALB/c nude mice (4 weeks old) were grown under standard conditions, containing a 12 h dark/light cycle, with 50% humidity and a 22 ± 2 °C room temperature, and water and standard pellet feed were freely available. HNE1 cells were treated with P8 fibrils for 3 days before injection. The mice were first randomly divided into two groups (*n* = 10). A total of 2 × 10^6^ HNE1 cells or P8 fibril-treated HNE1 cells mixed with 50% Matrigel (Corning Incorporated, Corning, USA) were injected subcutaneously into BALB/c nude mice. When the tumor volume reached approximately 150 mm^3^ (approximately 14 days after inoculation), each group of mice was randomly divided into 2 groups (*n* = 5) and intraperitoneally injected with 17-AAG (50 mg/kg) or an equal volume of PBS every other day. 17-AAG was formulated to 10 mg/mL in solvent containing 95% corn oil and 5% DMSO. Perfusion was continued for 3 weeks. Tumors were measured every three days, and the tumor volumes were measured with calipers for the length (L) and width (W) of the tumor according to the formula (L × W^2^)/2. To characterize the tumors, they were excised and embedded in paraffin before being sectioned.

### HE and IHC staining

A gradient ethanol series was used to rehydrate paraffin-embedded tissue sections from NPC patients and mouse xenografts for IHC staining. Endogenous peroxidases were blocked by hydrogen peroxide (0.3%). The sections were blocked with goat serum for 30 min, incubated with primary antibodies overnight at 4 °C, and finally incubated with secondary antibodies for 1 h at room temperature. DAB chromogen (Gene Tech, Shanghai, China) was used to stain the sections, which were subsequently counterstained with hematoxylin. According to the manufacturer’s instructions, dewaxed sections were stained with hematoxylin and eosin for HE staining.

### Transcriptome sequencing

RNA sequencing was performed by OEbiotech (Shanghai, China). Briefly, total RNA was extracted from C666-1 cells, C666-1 cells treated with P8 (C666-1-P8) and C666-1 cells infected with p53-R280T lentivirus and treated with P8 (C666-1-R280T-P8) using TRIzol reagent. A VAHTS universal V6 RNA-seq library prep kit (Vazyme) was used to construct the RNA library, followed by sequencing on an Illumina NovaSeqTM 6000 platform (Illumina, CA, USA). The differentially expressed genes (DEGs) were identified based on |Fold Change | >1.5 and *p* value < 0.05, and differential expression analysis was performed using DESeq2. Gene Ontology (GO) and Kyoto Encyclopedia of Genes and Genomes (KEGG) pathway enrichment analyses were performed to determine the significantly enriched terms through R (v3.2.0). Gene set enrichment analysis (GSEA) was carried out using GSEA software.

### Hsp90-p53 docking model

The Hsp90-p53 docking model was built by the HADDOCK web server. The ambiguous interaction restraints (AIR) parameter was set according to the NMR data [30]. The templates used in the docking program were the crystal structure of the yeast Hsp90 dimer (PDB ID 2CG9) and the crystal structure of the p53-DBD template p53 (PDB ID 2CG9).

### Statistical analysis

Statistical analysis was performed with GraphPad Prism 8.0 software (San Diego). Three times were represented by all quantified data. Data are represented as mean ± standard deviation (SD). The differences between two groups were performed with the two-tailed Student’s unpaired *t* test. The differences between more than two groups were performed with one-way ANOVA with Tukey’s multiple comparison test. There were statistically significant differences between the groups, *P < 0.05, **P < 0.01, ***P < 0.001, ****P < 0.0001.

### Supplementary information

Supplementary Figures

Supplementary Table 1

Supplementary Table 2

Original Data File

Reproducibility Checklist

## Data Availability

The datasets used and analyzed during the current study are included within the article and available from the corresponding authors upon reasonable request.
