# Supplementary material for: Amyloid aggregates induced by the p53-R280T mutation lead to loss of p53 function in nasopharyngeal carcinoma
Source: Cell Death Dis. 2024 Jan 11;15(1):35. doi: 10.1038/s41419-024-06429-8 (PMC10784298; doi:10.1038/s41419-024-06429-8)
Supplement: Supplementary file 1 — Supplementary Figures [file 41419_2024_6429_MOESM1_ESM.docx]

**Supplementary Figures:**

**
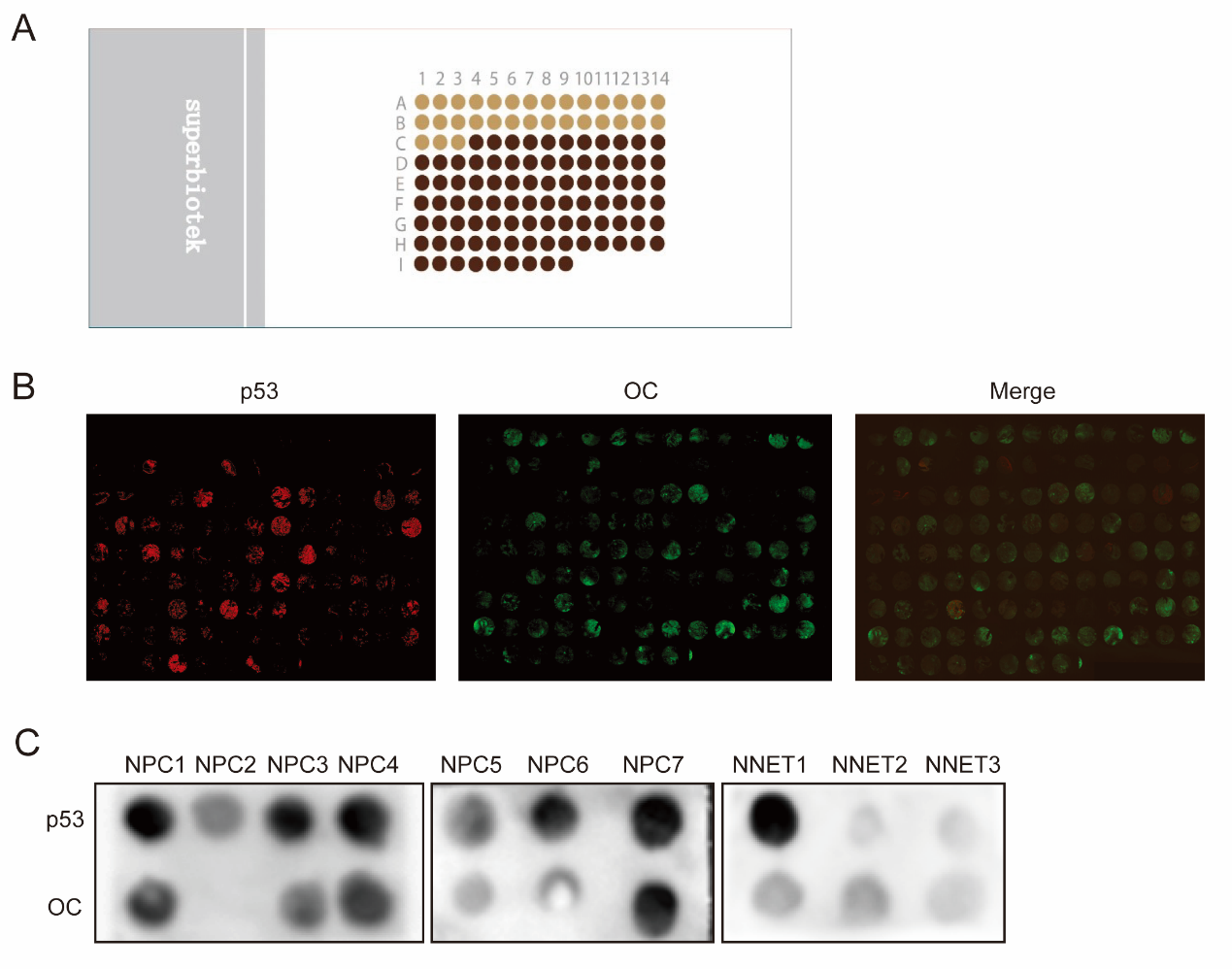
**

**Supplementary Fig. 1 Aggregated p53 amyloid in the NPC tissue array and TAF.** (A) Sample distribution in the tissue microarray. The pattern diagram for the type of samples on the microarray. A1 to C3 show samples of NNET tissues and are indicated by yellow circles. C4 to I9 are samples of undifferentiated nonkeratinizing NPC tissues and are indicated by brown cycles. (B) Immunofluorescence analysis of p53 aggregation in a tissue microarray containing NPC and NNET tissues. Red represents p53 staining, and green represents OC (specific to amyloids) antibody staining. (C) Dot blot results for TAF extracted from the other 7 cases of NPC and 3 cases of NNET tissues. NPC, nasopharyngeal carcinoma; NNET, normal nasopharyngeal epithelial tissues; TAF, tissue amyloid fraction.

**
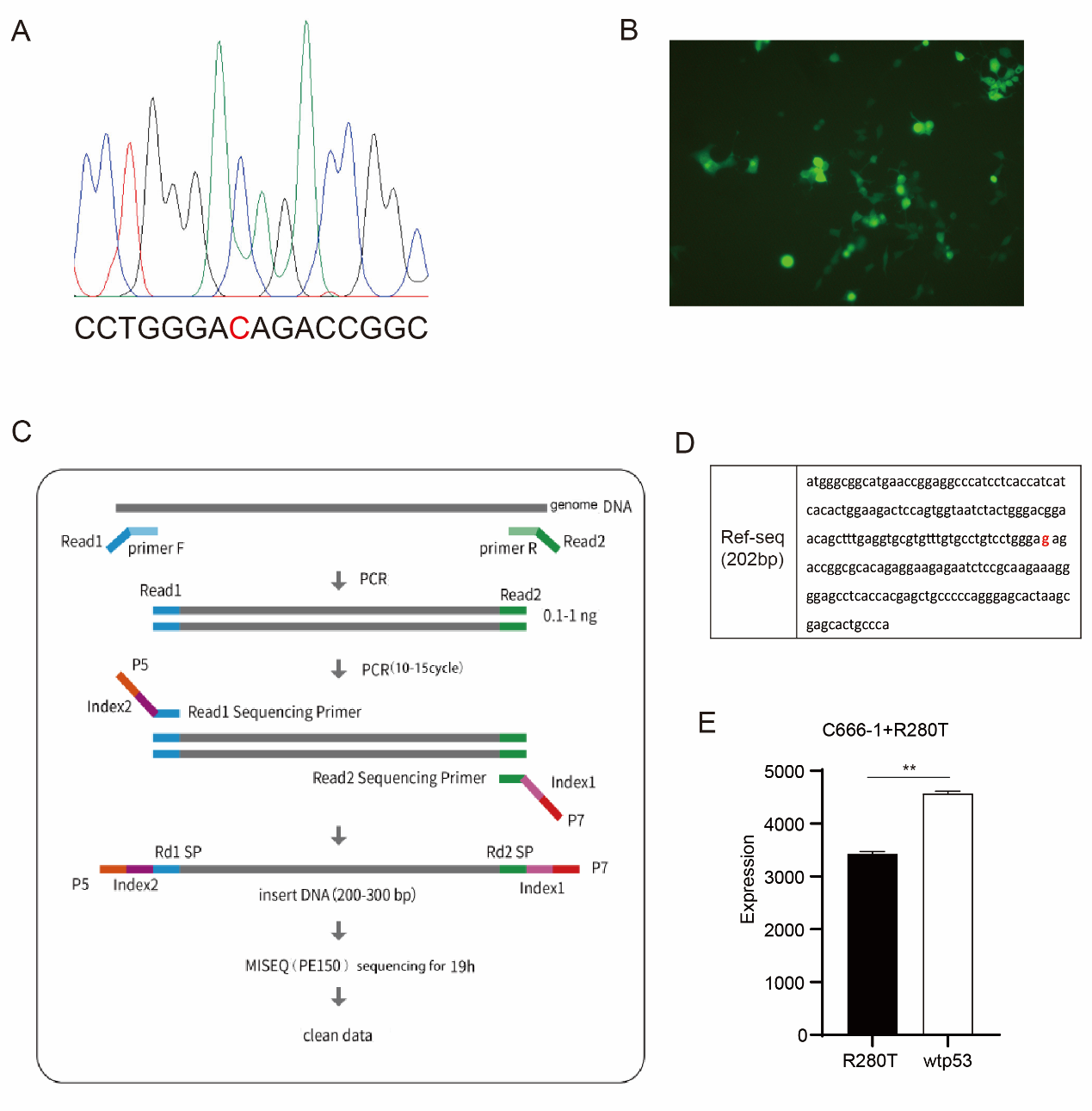
**

**Supplementary Fig. 2** **C666-1 cells were infected with lentivirus carrying the p53-R280T mutation.** (A) The pCDH-CMV plasmid expressing the R280T mutation was confirmed by sequencing. (B) Representative fluorescence images showing successful infection of p53-R280T lentivirus into C666-1 cells. (C) Schematic workflow showing fastNGS for infected C666-1 cells. (D) The reference sequence designed for fastNGS. (E) The p53R280T expression levels in comparison to endogenous p53 levels in C666-1 cells infected with R280T lentivirus. Data are represented as the mean ± SD. ***P* < 0.01


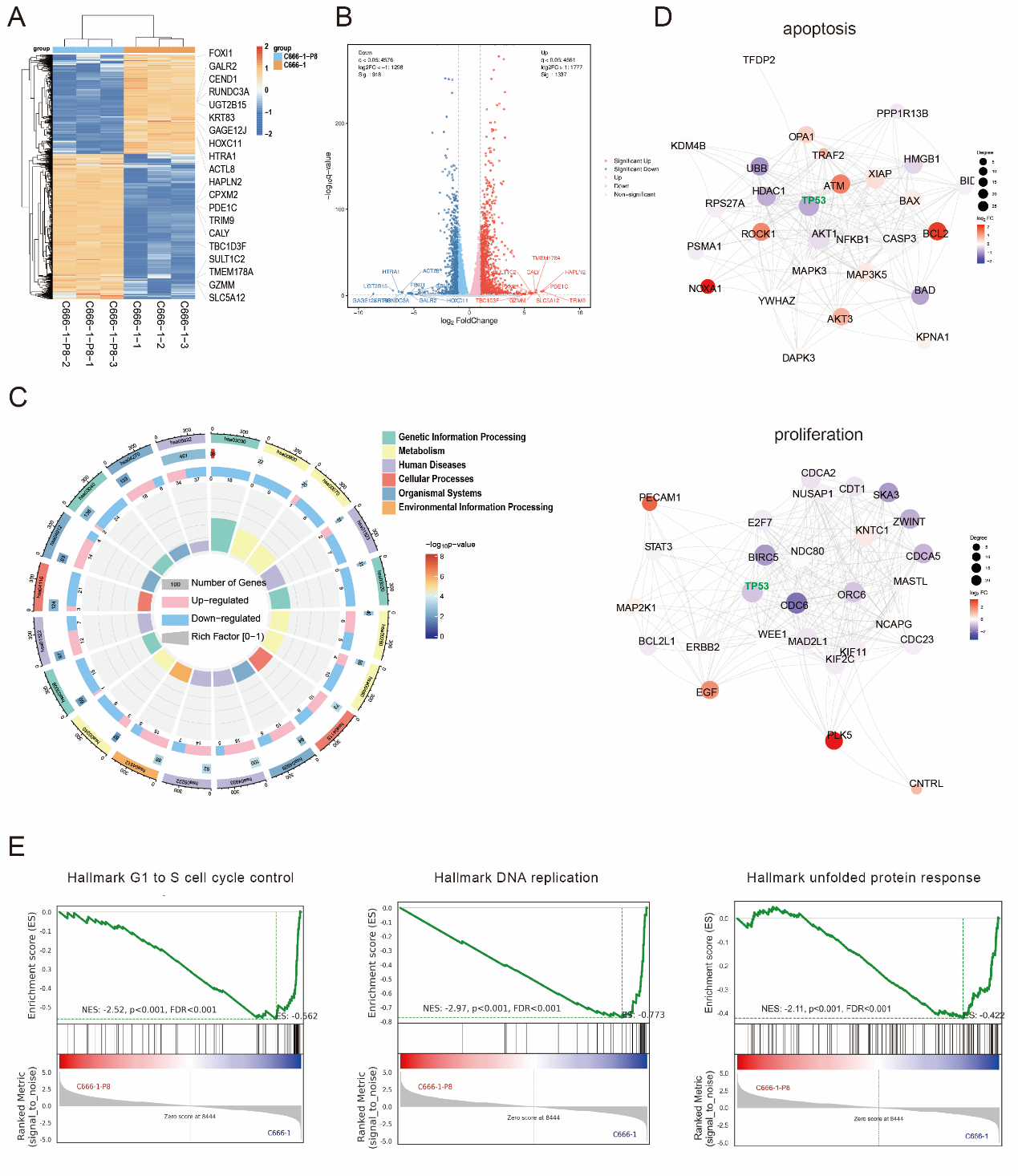


**Supplementary Fig. 3 T****ranscriptome analysis of C666-1 cells treated with P8 fibrils.** (A) A heatmap of differentially expressed genes based on cluster analysis. Orange and blue indicate relatively high and low expression between C666-1-P8 and C666-1 cells, respectively. (B) Volcano map of differentially expressed genes between C666-1-P8 and C666-1 cells. Red and blue represent the significantly upregulated and downregulated genes, respectively. Gray represents nonsignificant differentially expressed genes. (C) Circle plot of GO analysis for the differentially expressed genes between C666-1-P8 and C666-1 cells. (D) PPI network analysis of apoptosis- and proliferation-related genes differentially regulated by p53 in C666-1 cells treated with P8 fibril. The red and blue bubbles represent the upregulated and downregulated p53 target genes, respectively. The size of the dots indicates the number of interacting genes. (E) GSEA of the cell cycle, DNA replication, and unfolded protein response in C666-1-P8 cells compared with C666-1 cells.
