# Supplementary material for: Amyloid aggregates induced by the p53-R280T mutation lead to loss of p53 function in nasopharyngeal carcinoma
Source: Cell Death Dis. 2024 Jan 11;15(1):35. doi: 10.1038/s41419-024-06429-8 (PMC10784298; doi:10.1038/s41419-024-06429-8)
Supplement: Supplementary file 3 — Supplementary Table 2 [file 41419_2024_6429_MOESM3_ESM.docx]

**Supplementary Table 2. List of primers used in this study**

| **Gene** | **Forward primer (5'-3')** | **Reverse primer (5'-3')** |
| --- | --- | --- |
| p53-1 | CCGCAGTCAGATCCTAGCG | GGGCACCACCACACTATGTC |
| p53-2 | GCTGCTCAGATAGCGATGGT | AGTCTGAGTCAGGCCCTTCT |
| p53-280 | AAAGGACAAGGGTGGTTGGG | CAAATGCCCCAATTGCAGGT |
| ChIP-p21 | GTGGCTCTGATTGGCTTTCTG | CTGAAAACAGGCAGCCCAAG |
| ChIP-BAX | TAATCCCAGCGCTTTGGAA | TGCAGAGACCTGGATCTAGCAA |
| ChIP-PUMA | GCGAGACTGTGGCCTTGTGT | CGTTCCAGGGTCCACAAAGT |
| ChIP-NOXA | CAGCGTTTGCAGATGGTCAA | CCCCGAAATTACTTCCTTACAAAA |
| ChIP-MDM2 | GGTTGACTCAGCTTTTCCTCTTG | GGAAAATGCATGGTTTAAATAGCC |
| p21 | GCGACTGTGATGCGCTAATG | GAAGGTAGAGCTTGGGCAGG |
| BAX | AACTGGACAGTAACATGGAG | AGTTGAAGTTGCCGTCAGAA |
| PUMA | ACCTCAACGCACAGTACGAG | TAAGGGCAGGAGTCCCATGA |
| NOXA | CGCAAGAACGCTCAACCGAG | TTTCTGCCGGAAGTTCAGTTTG |
| β-actin | AGACGTGGACATCCGCAAAG | CTGGAAGGTGGACAGCGAGG |
| p53-reads | TCGTCGGCAGCGTCAGATGTGTATAAGAGACAGATGGGCGGCATGAACCG | GTCTCGTGGGCTCGGAGATGTGTATAAGAGACAGTGGGCAGTGCTCGCTTAGTG |
