# Supplementary material for: Amyloid aggregates induced by the p53-R280T mutation lead to loss of p53 function in nasopharyngeal carcinoma
Source: Cell Death Dis. 2024 Jan 11;15(1):35. doi: 10.1038/s41419-024-06429-8 (PMC10784298; doi:10.1038/s41419-024-06429-8)

1. Full and uncropped western blot for Figure 2B

Lanes 5,6,7,8 are on the figure


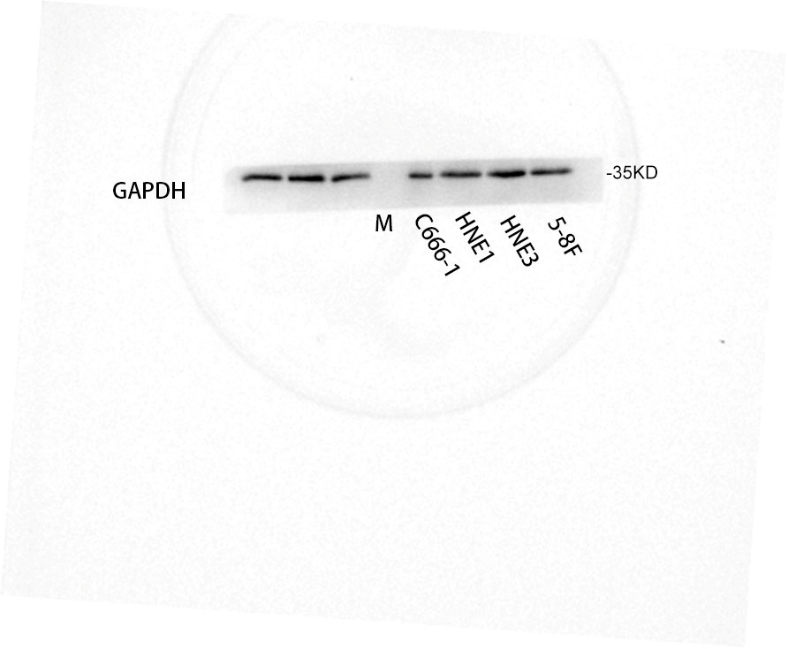


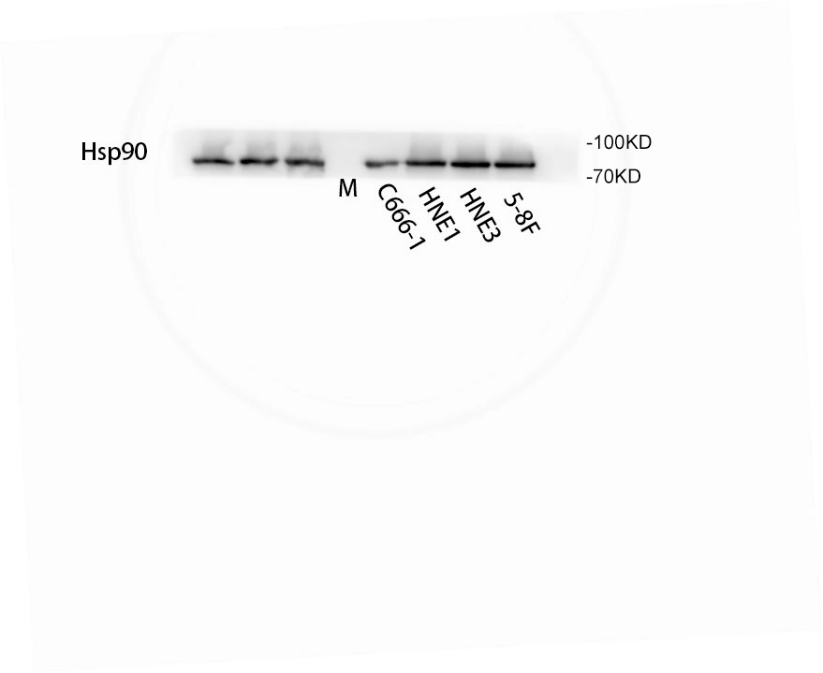


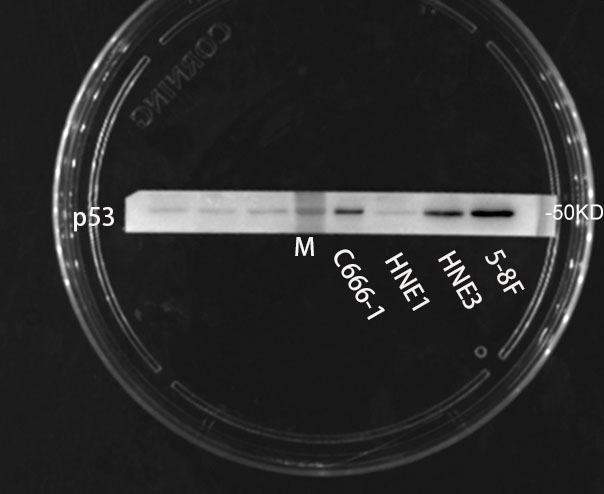


1. Full and uncropped western blot for Figure 2F

**
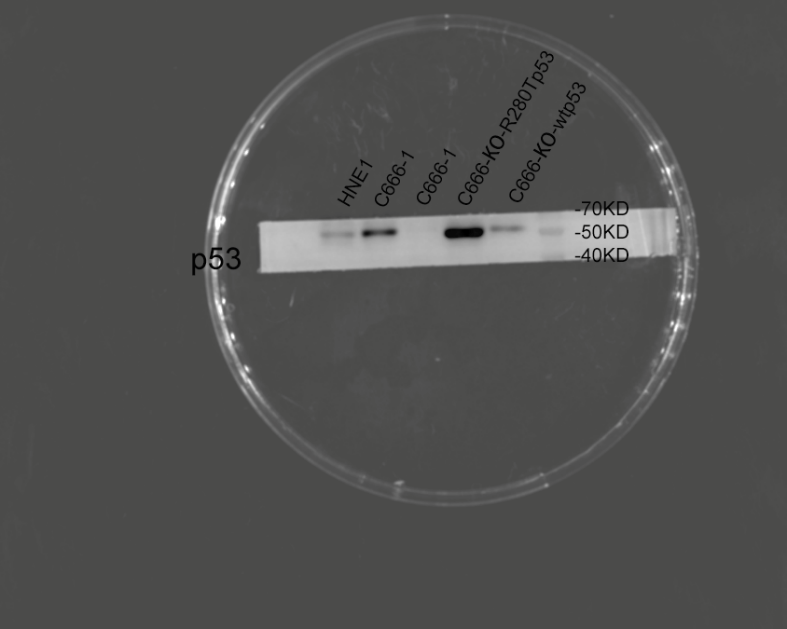
**

**
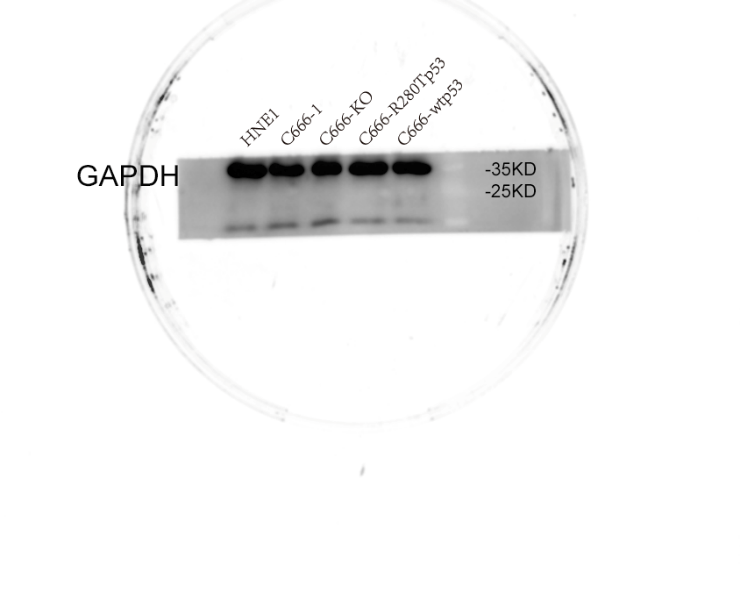
**

1. Full and uncropped western blot for Figure 3A


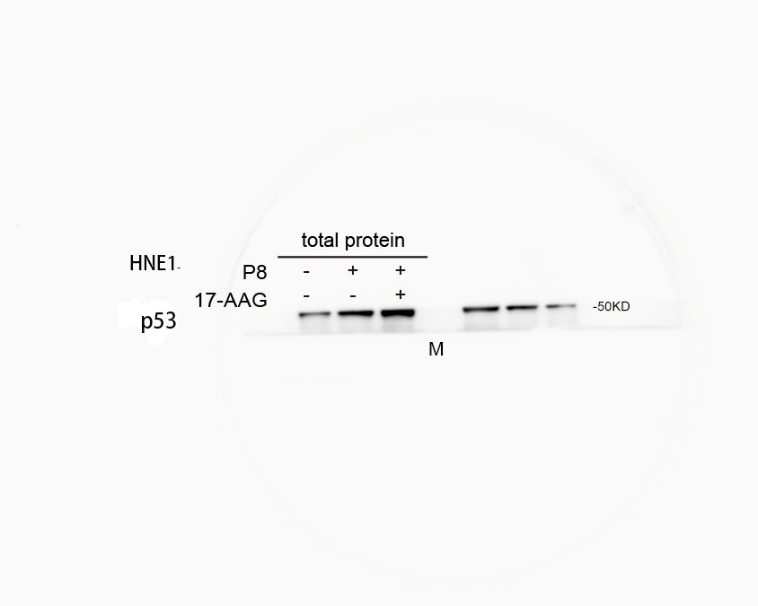


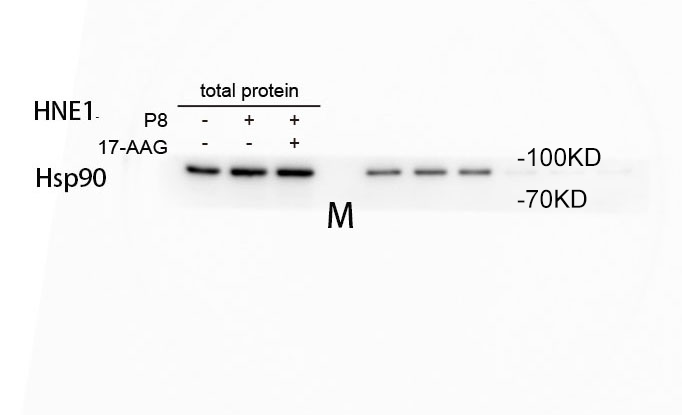

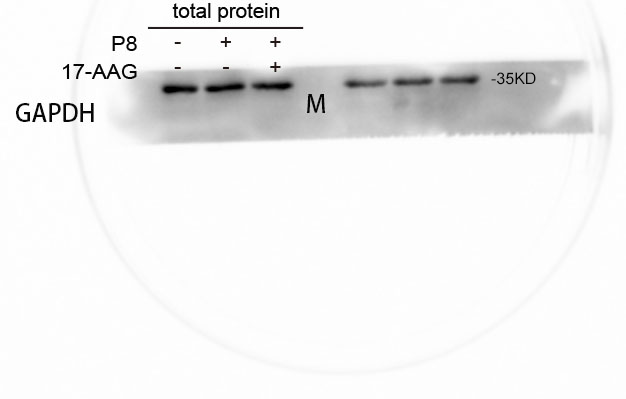


Lanes 5,6,7,8,9.10 are on the figure


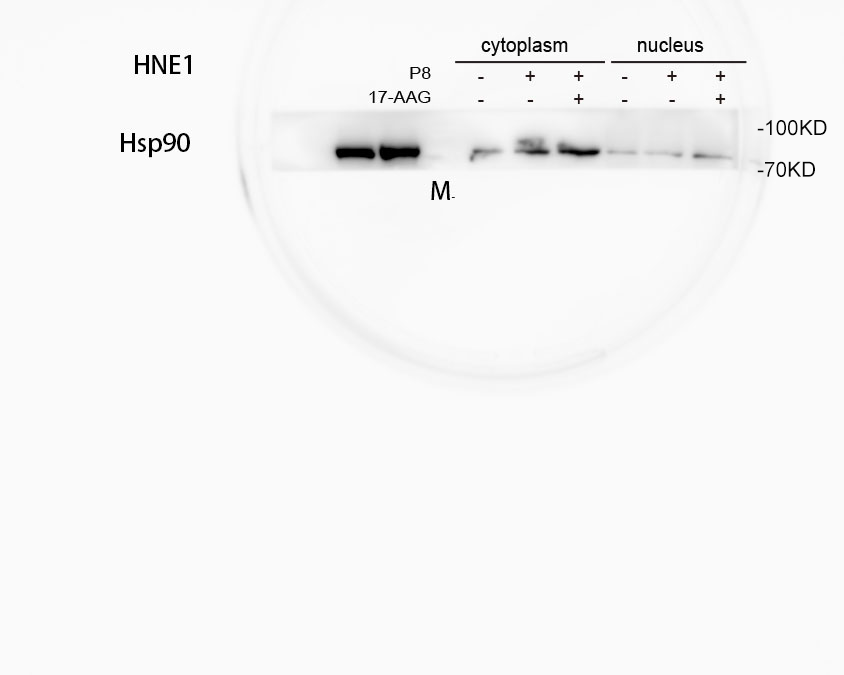

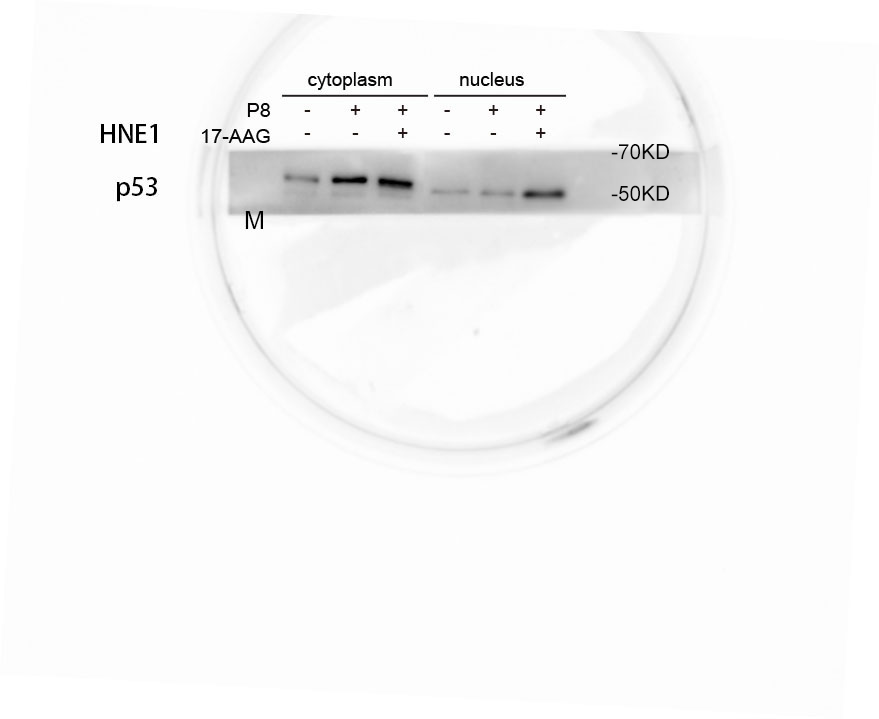


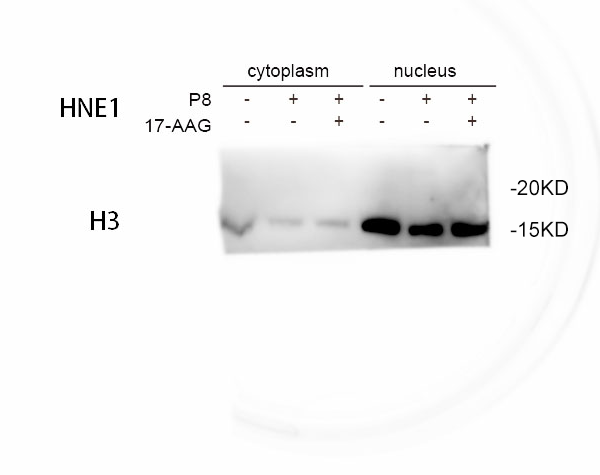

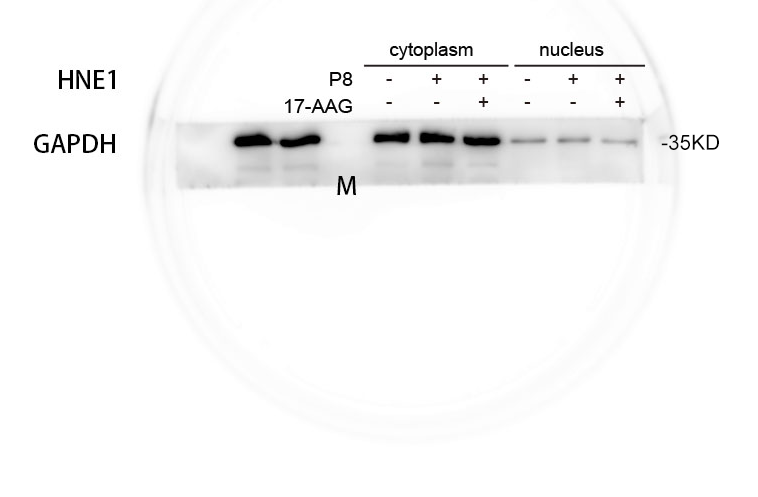


1. Full and uncropped western blot for Figure 3B


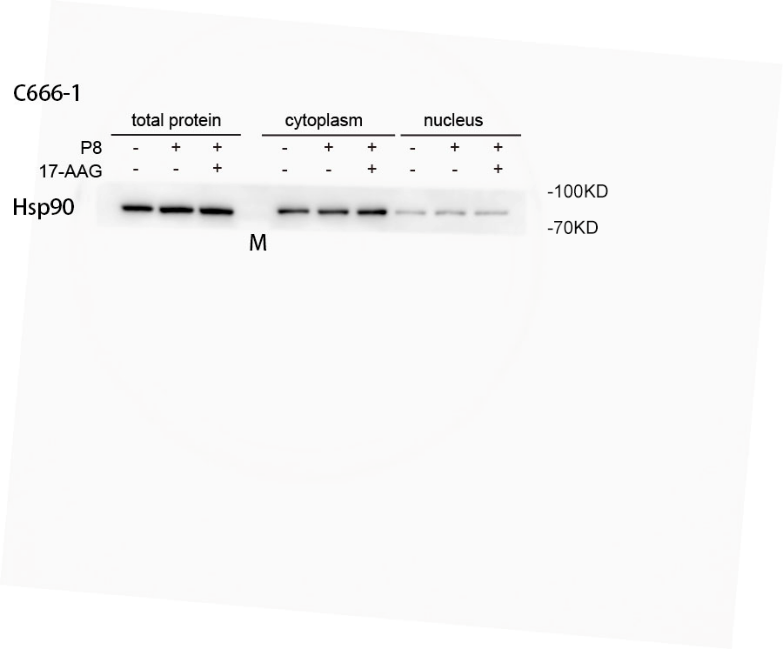


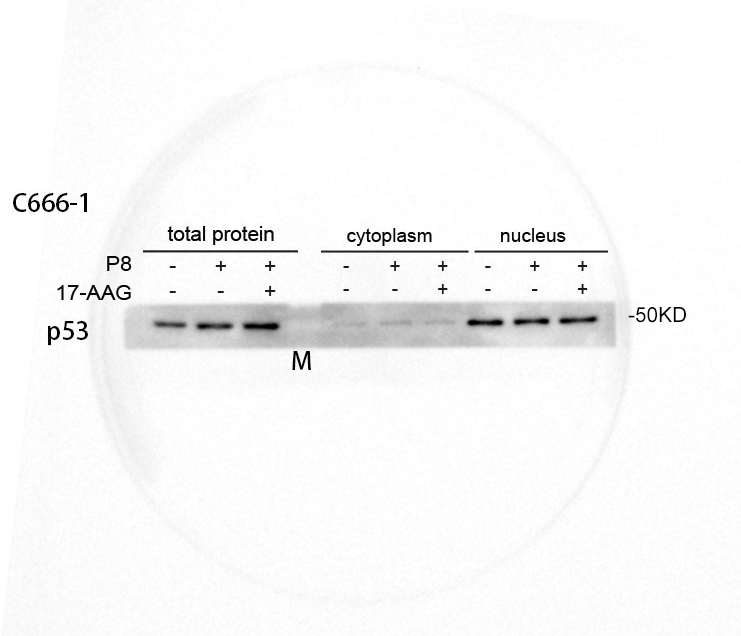


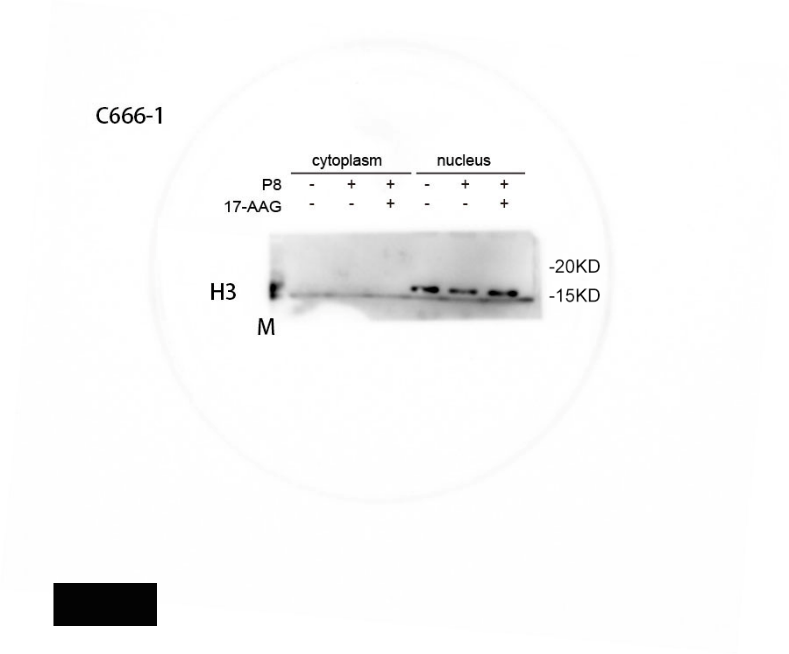


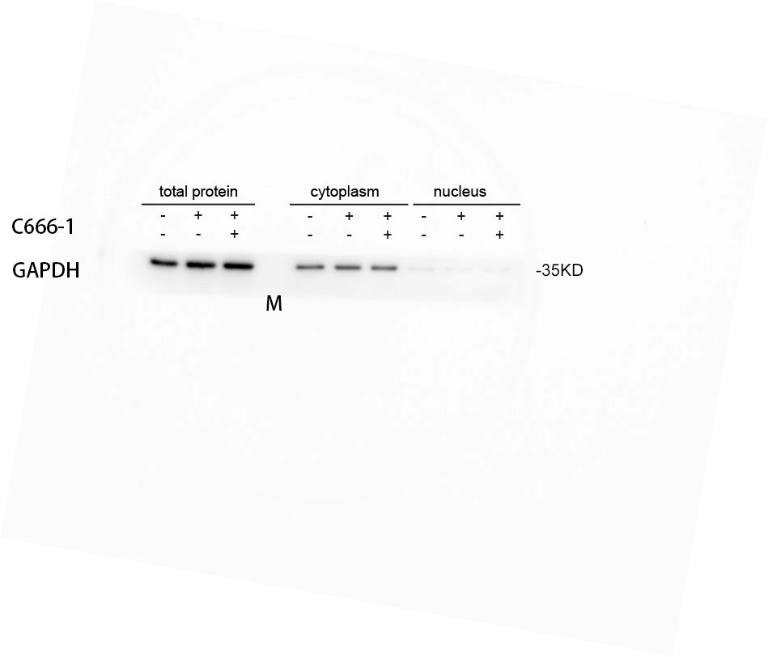


1. Full and uncropped western blot for Figure 4G

HNE1 cells: Lanes 2,3,4,5 are on the figure


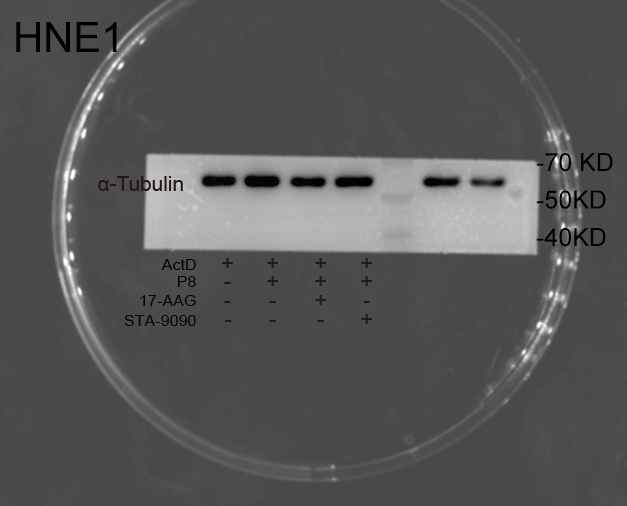

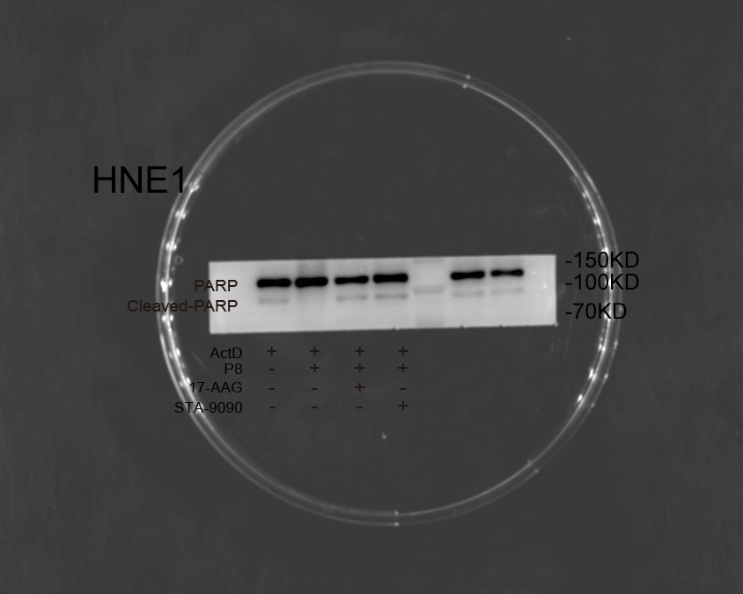

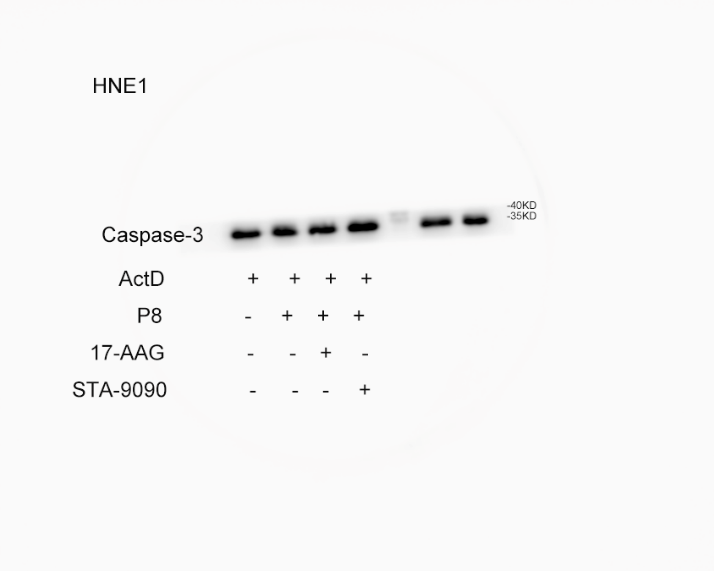


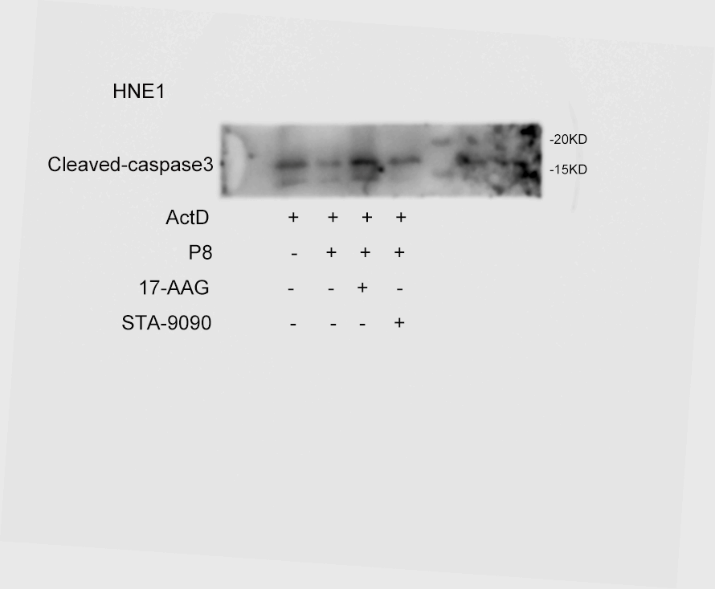


C666-KO-R280T: Lanes 2,3,4,5 are on the figure


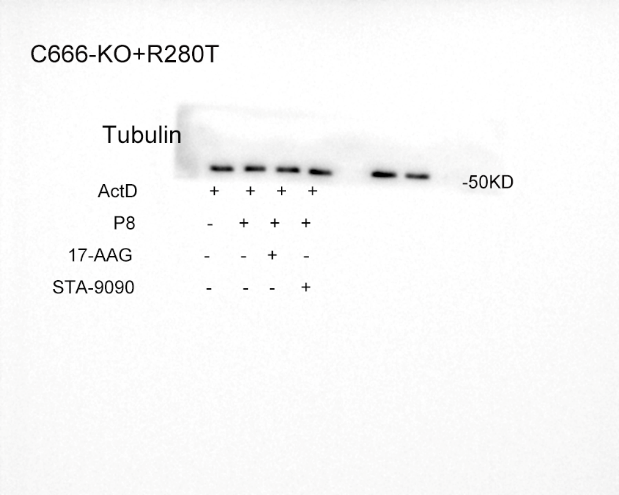


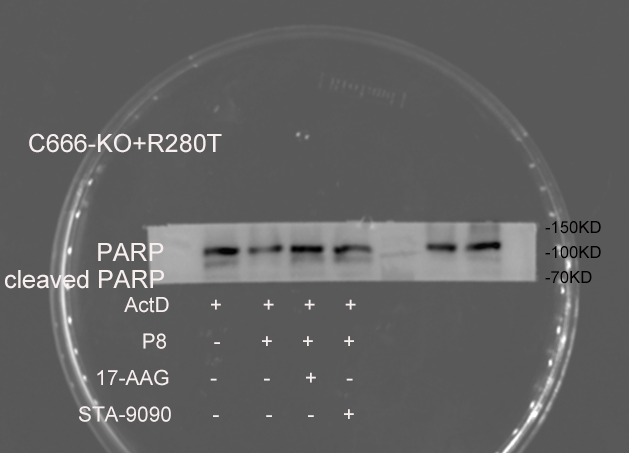


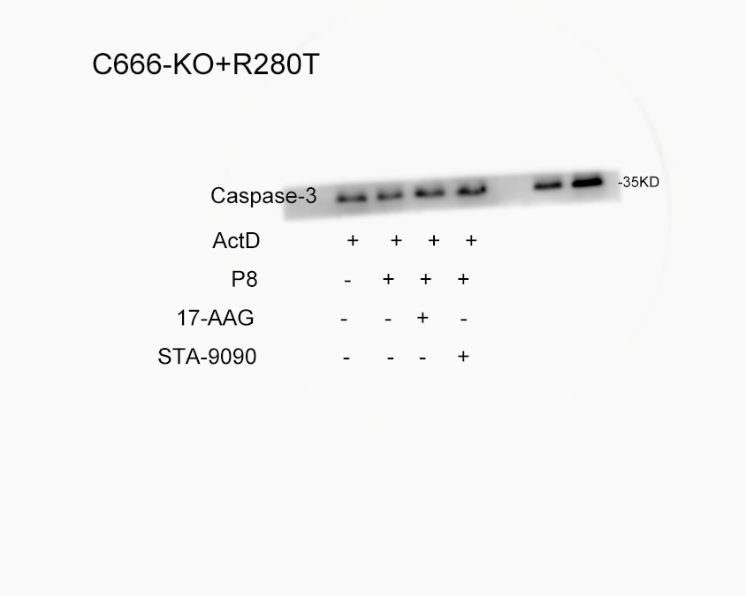


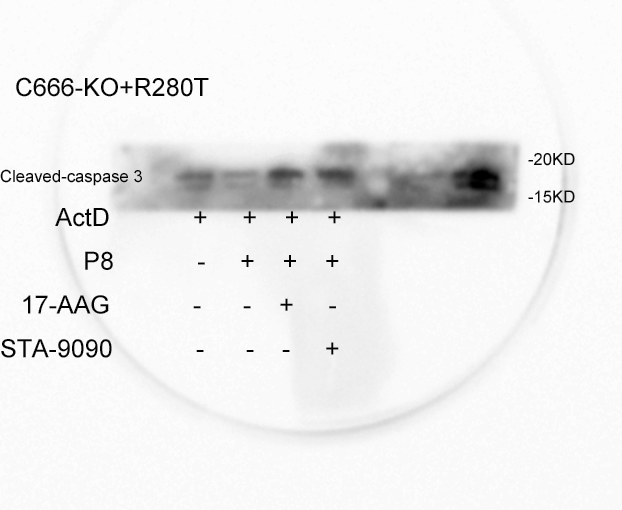


C666-1: Lanes1,2,3,4,5 are on the figure


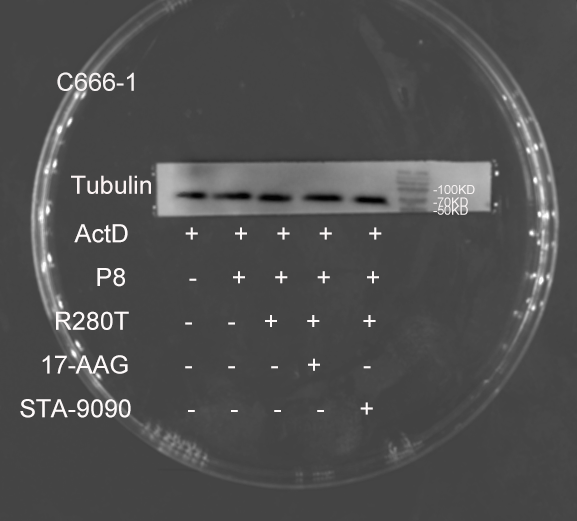


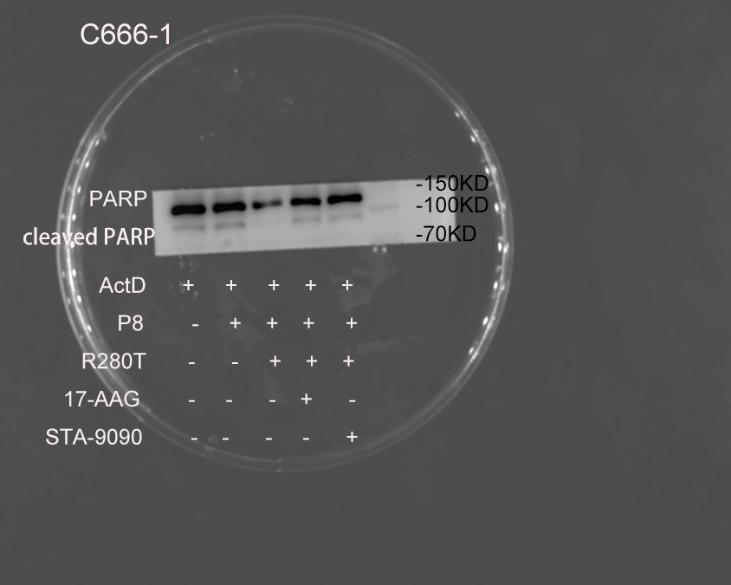


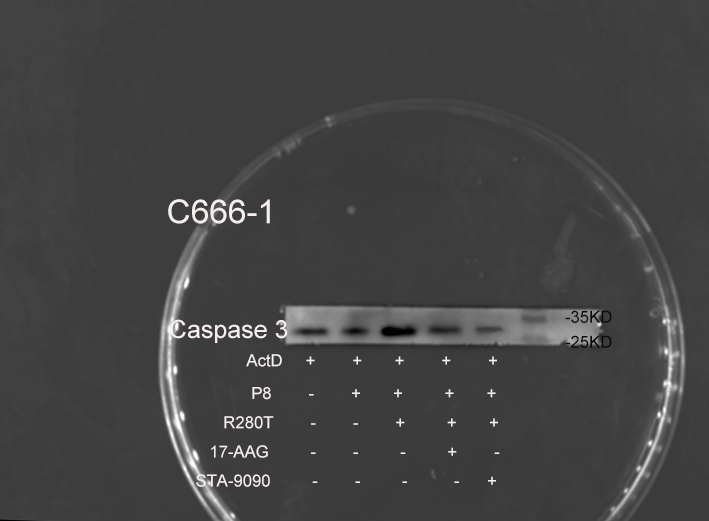


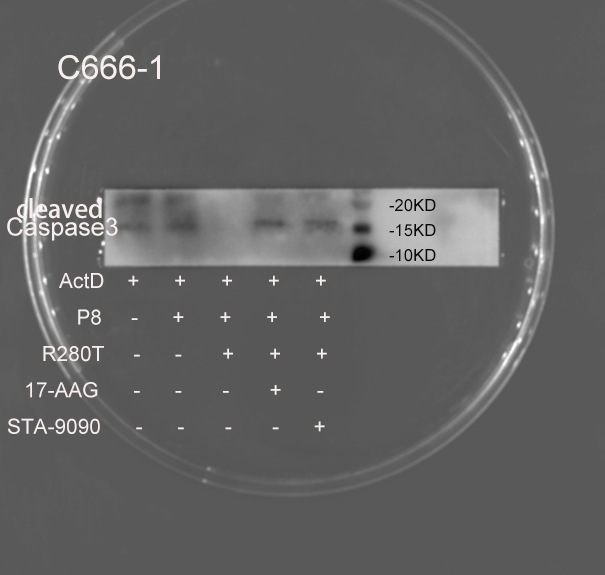

Supplement: Supplementary file 4 — Original Data File [file 41419_2024_6429_MOESM4_ESM.docx]
